# Metabolism dynamics in tropical cockroach during a cold-induced recovery period

**DOI:** 10.1186/s40659-025-00621-6

**Published:** 2025-06-14

**Authors:** S. Chowański, J. Lubawy, E. Paluch-Lubawa, M. Gołębiowski, H. Colinet, M. Słocińska

**Affiliations:** 1 Department of Animal Physiology and Developmental Biology, Faculty of Biology, Adam Mickiewicz University, Poznań, Poland; 2 Department of Plant Physiology, Faculty of Biology, Adam Mickiewicz University, Poznań, Poland; 3 Laboratory of Analysis of Natural Compounds, Department of Environmental Analysis, Faculty of Chemistry, University of Gdańsk, Gdańsk, Poland; 4 University of Rennes, CNRS, ECOBIO [(Ecosystèmes, biodiversité, évolution)] - UMR, Rennes, France

**Keywords:** *Gromphadorinha coquereliana*, Cold stress, Metabolic adjustments, Insect

## Abstract

**Background:**

Insects are poikilothermic organisms, meaning their body heat comes entirely from their surroundings. This influences their metabolism, growth, development, and behavior. Cold tolerance is considered an important factor in determining the geographic distribution of insects. The tropical cockroach *Gromphadorhina coquereliana* is capable of surviving exposure to cold. To determine the dynamics of metabolic adjustments occurring in the insect body under cold stress, we subjected the cockroach to 4°C for 3 h, followed by recovery periods of 3, 8, and 24 h. We then determined the levels of glycogen, proteins, lipids, amino acids, and carbohydrates. We also measured gene expression and the activity of the main enzymes of metabolic cycles responsible for energy conversion, namely, phosphofructokinase (PFK), hydroxyacyl-CoA dehydrogenase (HADH), and lactic acid dehydrogenase (LDH). All these analyses were conducted in different tissues: hemolymph, fat body, and muscle.

**Results:**

Our results show that in the fat body, protein degradation and an increase in unsaturated fatty acids (UFA) and cholesterol are observed, which likely allows membranes to maintain their functions. The high levels of lactic acid and LDH expression and activity indicate that anaerobic metabolic pathways are triggered. In the hemolymph, cold stress induces an increase in the levels of cryoprotective substances such as amino acids and sugars, which can also be used as a source of energy. On the other hand, muscle metabolism slows down (LDH, HADH), except for an increase in glucose, which may result from the gluconeogenesis process. During the recovery period, increased activity and expression of LDH, PFK, and HADH, as well as increased levels of UFA, lactic acid, glycerol, and TAG, were observed in fat body tissue, while in the hemolymph, increased levels of cryoprotectants still occurred.

**Conclusions:**

*G. coquereliana* shows partial freeze tolerance, combining traits of both freeze-intolerant and freeze-tolerant insects. This adaptation helps it survive brief cold periods and suggests an evolutionary move towards complete freeze tolerance. Although cold stress challenges *G. coquereliana* in maintaining metabolic homeostasis, these insects exhibit deep biochemical adjustments to cope with adverse environmental stressors such as low temperature.

**Supplementary Information:**

The online version contains supplementary material available at 10.1186/s40659-025-00621-6.

## Introduction

Many environmental factors challenge organism survival. One of them is temperature which strongly determines the range of species distribution, especially poikilothermic (ecothermic) animals such as insects. It means that their body heat comes from their surroundings and have reduced ability to thermo-regulate their body temperatures under varying temperatures, what influences their metabolism, growth, development, and behaviour [1]. Due to that insects have developed many strategies to survive in unfavorable low temperatures [2–4]. The strategies vary significantly among them, but the following groups of insects can be distinguished: (1) freeze-tolerant, (2) freeze-avoiding, and (3) chill-susceptible. Insects utilizing the first strategy can tolerate and survive the freezing of their tissues and body. Freeze-avoiding species lower the freezing point of their extracellular compartments by supercooling, which involves the accumulation of cryoprotectants to withstand temperatures below the freezing point of water. Chill-susceptible insects, on the other hand, die from cold-induced injuries before ice formation occurs within their bodies. Some species can also be referred to as chill-tolerant, depending on their relative sensitivity to low temperatures compared to chill-susceptible insects [5–8].

Insects adapt to cold stress by modulating main metabolic pathways [9–12], accumulating cryoprotectants, and synthesizing various proteins such as ice nucleating agents (INAs), antifreeze proteins (AFPs), and heat shock proteins (HSPs). Some adaptations delay the appearance of ice crystals, others prevent damage, and others help reduce the harmful effects of low temperature. As a result of cold stress, changes governed by the neuroendocrine system [13] in the metabolism of carbohydrates [14], lipids [15], and amino acids [16] can occur.

An important defence against cold stress is the accumulation of cryoprotectants, which allows for a decrease in the supercooling point (SCP) and helps avoid harmful ice formation [17]. In the gall moth *Epiblema scudderian*, body fluid can be supercooled to −40˚C [18, 19]. Increased levels of low molecular weight molecules in response to cold stress have been observed in many insect species [18], for example, in overwintering adults firebug *Pyrrhocoris apterus* [20], in the red palm weevil pre-pupa, *Rhynchophorus ferrugineus*, [21], in the flies *Drosophila immigrans* [22] and *Sitodiplosis mosellana* [23], and in the beetle, *Dendroctonus ponderosae* [24]. The synthesis of cryoprotectants is related to increased activity of glycolysis, gluconeogenesis and pentose phosphate pathway enzymes [11]. In the fly, *Sarcophaga bullata*, increases in phosphoenolpyruvate carboxykinase [25], glucose 6-phosphatase and fructose 6-phosphatase activity were observed during glycolysis and gluconeogenesis in response to rapid cold hardening [12]. Moreover, exposure to cold stress increases the activity of glycogen phosphorylase, providing a source of glucose from its storage form [26].

Cold stress response is closely tied to changes in lipid composition [27], both storage and cell membrane lipids [28], as seen in various insects, e.g., in the *Belgica antarctica* [29], Drosophilid flies [15, 30], and others [31, 32]. These changes include membrane adaptation, an increase in unsaturated fatty acids and a shift in phospholipid proportions, helping to keep it proper fluidity and usage them as a source of energy. Studies carried out on *P. apterus* [33] and the firebug *Pringleophaga marioni* moth [34] showed that cold stress affects the ratios of saturated to unsaturated and long-chain to short-chain fatty acids, and leading to increased lipid content post-cold stress.

The synthesis of cryoprotectants and other cryoprotective processes, as well as damage repair processes, are energy (ATP) consuming. Therefore, maintaining energetic homeostasis is an important aspect of the response to cold stress. Oxidative phosphorylation provides more ATP for cells than anaerobic processes; however, the ability to produce energy is significantly affected by cold stress and varies depending on the conditions of the cold stress and species-specific strategies [4].

When considering cold resistance, the resistance of insects from polar or temperate zones mainly comes to mind. Nevertheless, insects from warm regions can also be exposed to low temperatures, often above zero degrees Celsius and/or above their freezing point. As mentioned above, among these insects, chill-susceptible (sensitive to low temperatures) and chill-tolerant insects can be distinguished [8]. A good example is the tropical Madagascar hissing cockroach *Gromphadorhina coquereliana*, which is usually not exposed to low temperatures in its environment. However, from time to time, it can be affected by temperatures around 4 °C. In the last 16 years, the temperature in July, the coldest month on Madagascar, in Antsirabe was on average 17 °C during the day and 7 °C during the night with lowest temperatures ranging from 2 °C to 6 °C (Supp. Mat., https://www.worldweatheronline.com/antsirabe-weather-averages/antananarivo/mg.aspx). The exposure is rather short (3–4 h) but can be repeated [35–37]. Our previous studies showed that *G. coquereliana* can survive exposure to such temperatures even for 8 h and repeated 3 times. Moreover, this insect is partially able to survive temperatures below its SCP [35–38]. So, at this point, we know that this species is quite resistant to low temperatures, but it remains unclear what adaptations and mechanisms determine this resistance.

Understanding how insects cope with cold stress is crucial, especially given the changing climate and the potential for pests to spread to new regions. Deciphering these mechanisms is not only important for managing pest populations [39] but also for predicting how insect distributions may shift in response to climate change. Thus, in the presented studies, we aimed to determine how the tropical cockroach, *Gromphadorhina coquereliana*, adjusts its metabolism during cold stress. Considering that hissing cockroaches are used commonly as an educational tool in classrooms, museums, zoos, feed for reptiles or just pets all over the World and the fact that they can be a host of moulds (eg. *Aspergillus niger*) which are associated with allergies and asthma in humans, especially children [40], it is important to understand their capabilities of inhabiting different niches and surviving cold. We tried to focus broadly on changes at the metabolite level and the activity of enzymes involved in metabolic pathways. This allows us to gain a broader perspective on how *G. coquereliana*, a representative of evolutionarily old insects dating back to the Carboniferous period over 300 million years ago [41], can adapt and survive under low temperatures despite naturally living in tropical zones. In contrast to our previous studies, here we analysed the changes over time directly after a single cold stress treatment (3 h at 4 °C) as well as during recovery at three time points: 3, 8, and 24 h after treatment to check the dynamics of metabolic activity. We employed tissue-specific metabolomics together with gene expression analyses of genes encoding main metabolic enzymes as well as their activity.

## Materials and methods

### Insects

Cockroaches (*Gromphadorhina coquereliana*) were obtained from a colony maintained at the Department of Animal Physiology and Developmental Biology, AMU. Insects were bred under constant conditions of temperature (28 ± 1 °C) and relative humidity (65 ± 5%) under a photoperiod of 8 h of light to 16 h of dark, with free access to food (lettuce, carrots, and powdered milk) and water provided ad libitum. In experiments, insects (3–4 individuals) were kept in plastic boxes (15 × 30 × 20 cm) with carrot and water, under the same conditions (control) or were treated with low temperature (4 °C) for 3 h once (denoted as cold). The samples of analyzed tissues were collected immediately after the cold stress or during the recovery periods of 3, 8, or 24 h (denoted as 3 h, 8 h, or 24 h recovery, respectively) after the cold stress. Throughout both the breeding and experimental phases, insects had continuous access to food and water.

### Collection of tissues

The tissue samples were isolated on fixed time points. After decapitation, the legs were cut off and used to isolate muscles, using microtweezers. Next, using microsurgical scissors, the cuticle was cut around sidewall to open insect body. The fat body was isolated with microtwizzers and Malpighian tubules and tracheoles were removed. The hemolymph, because of its short time of coagulation (15–20 s), also was isolated with microtweezers. Isolated tissue samples were immediately placed in 1.5 ml Eppendor’s tube and kept on the ice to avoid degradation of proteins and analysed metabolites by enzymes. Next, the samples were prepared specifically for analysed parameters.

### Determination of triacylglycerides and free glycerol

The triacylglycerides (TAG) and free glycerol levels were determined by spectrophotometric measurements according to modified procedure described previously by Tennessen*, *et al*.* [42]. Tissues, after isolation, were dried to a stable weight at 60 °C under a vacuum (−0.9 atm) and weighed in order to determine the dry weight of the samples. One biological sample was pooled from three insects. Then, samples were homogenized in 500 μl of PBS with 0.05% Tween buffer (PBS-T) and vortexed for 5 min at room temperature (RT). Next, they were centrifuged for 10 min at 1,000 g at RT to obtain supernatant, which was transferred to clean tubes. Subsequently, the supernatant was heated at 70 °C for 5 min and snap frozen in liquid nitrogen. The samples prepared in this way were stored for further analysis at −80 °C. On the analysis day, two tubes (A and B) were prepared for each sample. Tube “A” was utilized to measure the level of free glycerol while in tube “B” the TAG was digested by lipase to free the glycerol. Next, 20 μL of sample were placed in the tubes and then to tube “A” 20 μL of PBS-T was added, while to tube “B” 20 μL of triglyceride reagent (Merck Sigma-Aldrich, Poznań, Poland; T2449) were added. Samples were then placed at 37 °C and incubated for one hour. After that time, the samples were centrifuged at 10,000 g for 3 min. 30 μL of each sample were placed in a 96-well plate and 100 μL of free glycerol reagent (Merck Sigma-Aldrich, Poznań, Poland; F6428) were added, and incubated for 10 min at 37 °C. Next, the absorbance of samples was measured at wavelength λ = 540 nm using Synergy H1 Hybrid MultiMode (BioTek, USA) plate reader. The level of TAG was determined by subtracting the absorbance of samples in tube “A” (free glycerol) from samples in tube “B” showing total glycerol level that have been obtained by incubating samples with triglyceride reagent. The glycerol triolein-equivalent (Merck Sigma-Aldrich, Poznań, Poland; G7793) was used to prepare standard curve. The content of TAG and free glycerol in the analysed tissues was expressed as μg of TAG/glycerol per mg of dry tissue. The analyses were conducted in 8 biological replicates, tissues collected from one insect constituted one biological replicate.

### Gas chromatography–mass spectrometry (GC–MS)

The analysis of free fatty acids was done according to the method described previously by Szymczak-Cendlak*, *et al*.* [43] and Słocińska*, *et al*.* [44] with some modification. After dissection, the samples were placed in 4 mL brown-glass vials. Each sample was pooled from tissues originating from four individual insects, and four biological replicates were conducted for control while for the other treatments three. After collection, the samples were dried and weighted to determine the dry mass of sample. Next, the analysed components were extracted in 30 mL of dichloromethane (P.P.H., Stanlab, Lublin, Poland). The gentle stream of nitrogen over samples was used to remove the solvent. Silylalization of extracted compounds was conducted by adding 100 µL of a mixture of *N*,*O*-Bis(trimethylsilyl)trifluoroacetamide with trimethylchlorosilane (Merck Sigma-Aldrich, Poznań, Poland; T6381) at 100 °C for 1 h on the day of analysis. Gas chromatography-mass spectrometry (GC–MS) measurements were performed as described previously [44].

### Carbohydrates analysis

For determination of glycogen and glucose level in fat body and muscles, the spectrophotometric method and enzymatic assay was used according to Tennessen, et al. [42] with some modification. The samples were prepared in the same way as in case of TAG and free glycerol analysis. To determine the level of free glucose a Glucose Oxidase (GO) assay kit (Merck Sigma-Aldrich, Poznań, Poland; GAGO-20) was used. 20 μL of samples were placed on 96-weel plate, mixed with 65 μL of GO reagent and left for incubation (37 °C, 60 min). After incubation, to stop the reaction, 65 μL of 12 N H_2_SO_4_ were added and incubated for 10 min. After that, the absorbance of samples was measured at wavelength λ = 540 nm with Synergy H1 Hybrid MultiMode (BioTek, USA) plate reader. For the standard curve, the glucose at concentration 0.12, 0.08, 0.04, 0.02 and 0.01 mg/mL was used. For glycogen, the same procedure was used with the difference that amyloglucosidase (Merck Sigma-Aldrich, Poznań, Poland; A1602) was added to GO reagent (1 μL of amyloglucosidase per 1 mL of GO reagent). For standard curve, the glycogen at concentration 0.01, 0.02, 0.04, 0.08, 0.10, 0.15, 0.20 mg/mL was used. The content of analysed carbohydrates in the analysed tissues was expressed as μg of carbohydrate per mg of dry tissue. The analyses were conducted in at least 8 biological replicates (For muscles: Control – 14; 3 h cold – 16; 3 h recovery – 8; 8 h recovery – 8; 24 h recovery – 8; and for Fat body: Control – 14; 3 h cold – 14; 3 h recovery – 8; 8 h recovery – 8; 24 h recovery – 8).

### Lactate Dehydrogenase (LDH) activity assay

The LDH activity was determined spectrophotometrically with an enzymatic kit (Merck Sigma-Aldrich, Poznań, Poland; MAK066). The tissues (fat body and muscles) from 3 individuals were isolated to ice-cold PBS and homogenized with Teflon homogenizer, such sample constituted one biological replicate. Next, the obtained homogenate was centrifuged for 20 min at 12,000 rpm in 4 °C. The supernatant was transferred to a new tube and centrifuged again for 10 min at 12,000 rpm in 4 °C. The supernatant was transferred to a new tube and 2 μL of it was used to measure the concertation of soluble proteins. The obtained samples were kept in −80 °C until analysis. On the day of analysis, all samples were diluted with cold PBS to obtain final concentration of 4 μg/μL of proteins. 50 μL of samples were placed on well in 96-well plate (kept on ice) and mixed with 50 μL of Master reaction Mix (48 μL of LDH assay buffer and 2 μL of LDH substrate mix). After 3 min of initial incubation in 37 °C, the absorbance at wavelength λ = 450 nm was measured in every 3 min. The measuring continued until the value of the most active sample exceeded the absorbance value for the highest standard of NADH (12.5 nmole/well). The LDH activity was calculated with the following formula:

$$LDH activity=\frac{\frac{B}{\text{reaction time }\times \text{ V}} }{protein\;concentration},$$

Where *B* = amount of NADH [nmole] generated between initial and final measurement calculated basing of standard curve for NADH; *reaction time* = t_final_ – t_initial_ [min]; *V* = sample volume added to the well. We used the following concentration of NADH for standard curve: 2.5, 5, 7.5, 10 and 12.5 nmole/well. The activity is expressed as mU/mL/mg of proteins. Each sample was pooled from tissues originating from three individual insects, and eight biological replicates were conducted for all treatments.

### Phosphofructokinase (PFK) activity assay

The PFK activity was determined spectrophotometrically with colorimetric enzymatic kit (Merck Sigma-Aldrich, Poznań, Poland; MAK093) according to manufacturer’s protocol with some modification. The activity was determined in the same samples as for LDH activity assay. On the day of analysis, all samples were diluted with cold assay buffer to final concentration of 4 μg/μL of proteins. Due to potential high background signal, each sample’s background absorbance was measured as a “blank sample”. 50 μL of each sample were placed on two wells of 96-well plate (kept on ice) and mixed, in case of samples, with 50 μL of Reaction Mix A (42 μl of PFK assay buffer, 2 μl of PFK enzyme mix, 2 μl of PFK developer, 2 μl of ATP solution and 2 μl of PFK substrate) or in case of “blank sample” with 50 μL of Reaction Mix B (44 μl of PFK assay buffer, 2 μl of PFK enzyme mix, 2 μl of PFK developer, 2 μl of ATP solution). Next, after 5 min of incubation at 37 °C, the initial absorbance at wavelength λ = 450 nm was measured. The measurements were repeated in every 3 min until the value of the most active sample exceeded the absorbance value for the highest standard of NADH (10 nmole/well). The PKF activity was calculated with the following formula:

$$PKF activity=\frac{\frac{B}{\text{reaction time }\times \text{ V}} }{protein\;concentration},$$

Where: *B* – amount of NADH [nmole] generated between t_initial_ and t_final_ (calculation based on standard curve); *reaction time* = t_final_ – t_initial_ [min]; *V* = sample volume added to the well [mL]. We used the following concentration of NADH for standard curve: 2.5, 5, 7.5 and 10 nmole/well. The activity is expressed as mU/mL/mg of proteins. Each sample was pooled from tissues originating from three individual insects, and eight biological replicates were conducted for all treatments.

### Hydroxyacyl-CoA dehydrogenase (HADH) activity assay

The activity of HADH was measured calorimetrically according to procedure described previously by Cheung*, *et al*.* [45] with some modification. The activity was determined in the same samples as for LDH and PFK. All samples were diluted with PBS to final concentration of proteins equals 4 μg/μL. In the next step, 10 μL of samples were transferred on wells of 96-well plate (kept on ice) and mixed with 137.5 μL of reaction mixture (125 μL of PBS (pH = 6.0), 2.5 μL of 7.5 mM NADH, and 10 μL of 34.4 mM EDTA). After shaking, to each sample 2.5 μL of 5.9 mM acetoacetylo-CoA were added. To blank, 2.5 μl of PBS instead of acetoacetylo-Co were added. After 5 min of incubation, the initial measurement of absorbance (at λ = 340 nm) was done, and measurement was repeated each minute for 15 or 30 min in case of muscles and fat body samples, respectively. The HADH activity was calculated with following formula:

$$HADH activity=\frac{V}{\varepsilon \times d \times v} \times \frac{\Delta E}{t},$$

Where: *V* — final volume of the solution in a well [mL]; *ε* — extinction coefficient of NADH (6.22 mmol^−1^ × cm^−1^ at 340 nm); *d* — optical length of the well [cm]; *v* — volume of sample added to well [mL]; *ΔE* — change in absorbance (between t_initial_ and t_final_); *t* — reaction time (t_initial_ – t_final_). The activity is expressed as U/mL/mg of proteins. Each sample was pooled from tissues originating from three individual insects, and eight biological replicates were conducted for all treatments.

### Soluble protein concentration

The Direct Detect (Merck Sigma-Aldrich, Poznań, Poland) infrared spectrometer was used for determination of soluble proteins concentration as described previously [46]. Briefly 2 μL of protein sample, from samples used for determination of enzyme activity, were placed onto a PTFE membrane (Merck Sigma-Aldrich, Poznań, Poland; DDAC00010), inserted into the apparatus, dried for 2 min, and then measured. The system uses Fourier-Transform Infrared (FT-IR) spectroscopy to measure the amide bonds in the protein chains. This method doesn’t rely on the protein’s amino acid composition or dye-binding properties. Each sample was pooled from tissues originating from three individual insects, and eight biological replicates were conducted for all treatments.

### Transcript level analysis

Isolated tissues were placed in 300 μL of RNA lysis buffer (Zymo Research, Irvine, CA, USA) and homogenized for 2 min using a pellet homogenizer (Kimble Chase, Vineland, NJ, USA). For all experimental variants, three biological replicates, each pooled from 3–4 insects, were used. After homogenization the samples were snap frozen in liquid nitrogen and stored in freezer at − 80 °C for subsequent analysis. To extract total RNA Insect RNA MicroPrep™Kit (Zymo Research Corp., Irvine, CA, USA) was used according to protocol provided by manufacturer. The protocol included in-column DNase I treatment in order to remove gDNA contamination. A ReverAid™ Reverse Transcriptase (Fermentas, Waltham, MA, USA) was used to synthetize cDNA used for Quantitative Real‐Time PCR (RT-qPCR), which was conducted using CFX Opus Real-Time PCR System (BioRad, Poland) with Sensitive RT HSPCR Mix SYBR® kit (A&A Biotechnology s.c, Poland). Each biological replicate was run in two technical repetitions and the expression level of *G. coquereliana 18S rRNA* was used as a reference. “No template” negative controls (DNA/RNA free water) were run together with samples in order to test for any potential contaminations. The 2^−ΔΔCt^ method was employed to calculate the relative expression of tested genes. The primers used are listed in Supplementary Table 1.

### Statistical analysis

For calculation of statistical differences between samples GraphPad Prism software ver. 9 (GraphPad Software, San Diego, CA, USA) was used. In the first step we checked for the normality of distribution using the Shapiro–Wilk test. For normally distributed data, a one-way ANOVA with Dunnett’s post hoc test or Student’s t-test was used to calculate the statistical differences between the control and samples from all treatments. For data without normal distribution, the Kruskal–Wallis test with Dunn’s post hoc test was employed. All data are expressed as mean values ± SD, with the number of replicates (n) specified. Differences were considered statistically significant at the following levels: *p* ≤ 0.05 (*), *p* ≤ 0.01 (**), *p* ≤ 0.001 (***) or *p* ≤ 0.0001 (****). For metabolites presented in the heat maps a principal component analysis (PCA) was performed using build in option of the GraphPad Prism.

## Results

### Changes in protein level after cold stress

**Fig. 1:**
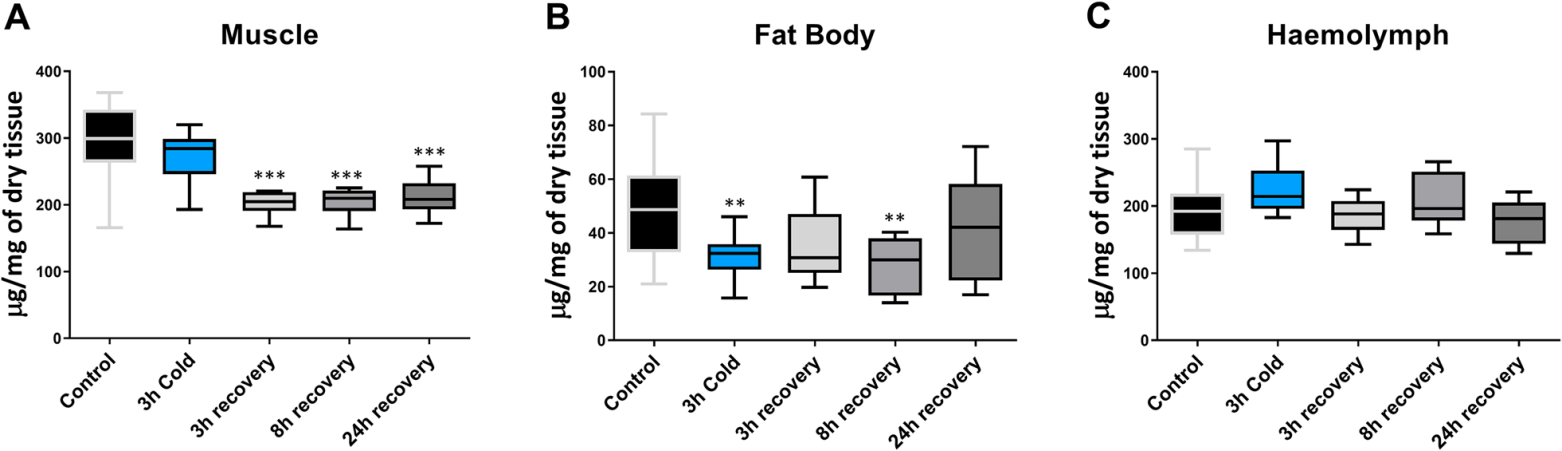
Changes in total protein content in muscles (**A**), fat body (**B**) and hemolymph (**C**) of *G. coquereliana* subjected to cold stress (4 °C) for 3 h and in recovery (3h, 8 h and 24 h) after cold stress. The centre line in boxes represent median value while the whiskers minimal and maximal value (*n* ≥ 8). Statistical significance to control is indicated by either *p* ≤ 0.01 (**) *p* ≤ 0.001 (***) or *p* ≤ 0.0001 (****). Statistical significance determined using One-way ANOVA with Dunnett’s multiple comparison test

In muscle tissue, we observed a significant decrease in protein levels (F = 13.02, *p* < 0.0001) after 3, 8, and 24 h of recovery following a single cold exposure. The levels dropped by 31% (Dunnet’s post-hoc, *p* < 0.0001), 30% (Dunnet’s post-hoc, *p* < 0.0001), and 30% (Dunnet’s post-hoc, *p* < 0.0001), respectively (Fig. 1A). However, no changes were noted during the exposure to stress itself. Treatments had significant effect on protein level in fat body (F = 3.871 *p* = 0.0085), where a significant decrease in protein levels was observed during cold stress (36%, Dunnet’s post-hoc, *p* = 0.0092) and after 8 h of recovery (42%, Dunnet’s post-hoc, *p* < 0.0083) (Fig. 1B). The protein level in the hemolymph did not change throughout the entire experiment (Fig. 1C), nor did the hydration of this tissue (data not shown).

### Changes in carbohydrates, amino acids, and glycogen level after cold stress

**Fig. 2:**
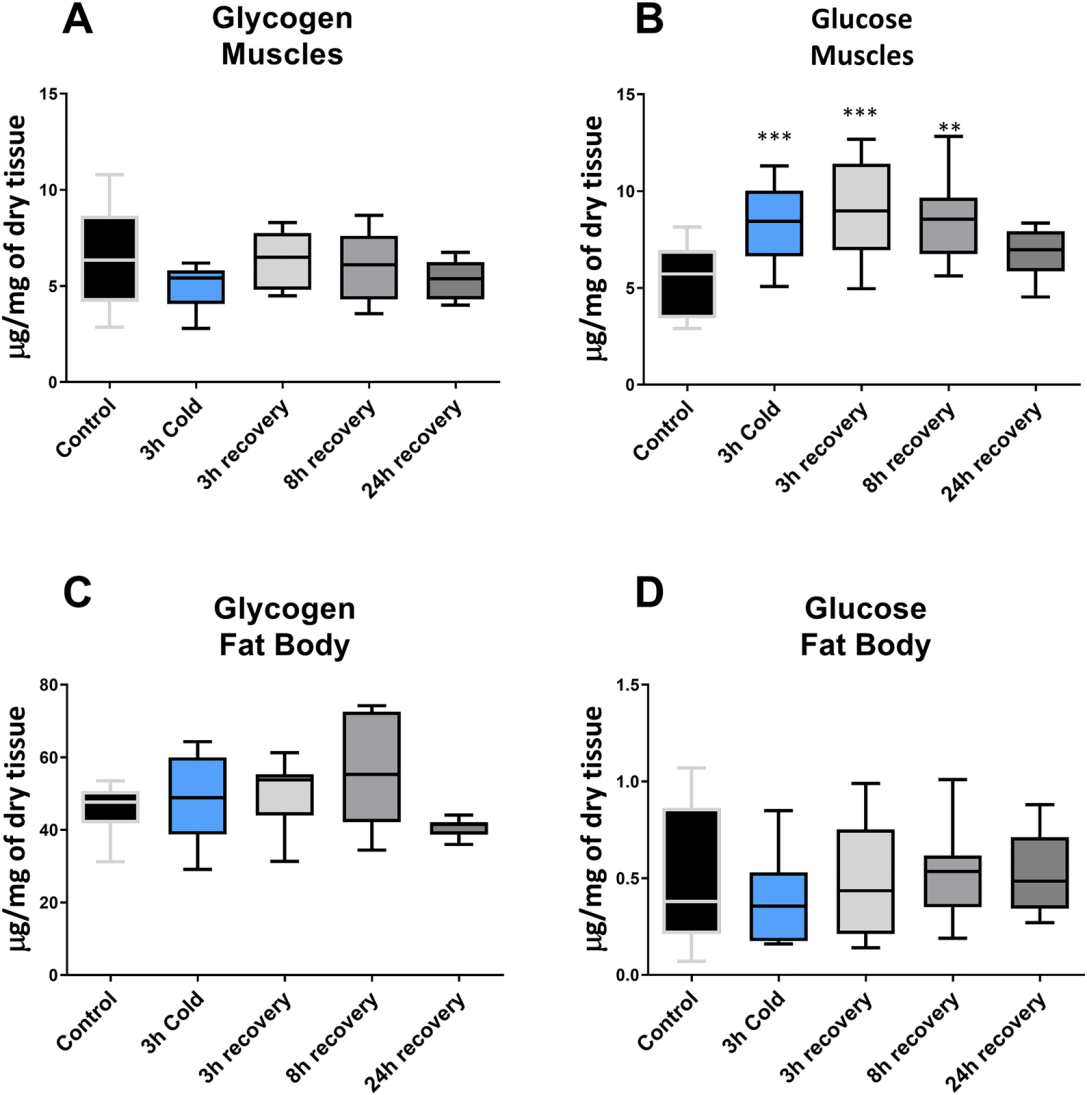
Changes in glycogen (**A**, **C**) and glucose (**B**, **D**) level in muscle (**A**, **B**) and fat body (**C**, **D**) of *G. coquereliana* subjected to cold stress (4 °C) for 3 h and in recovery (3h, 8 h and 24 h) after cold stress. The centre line in boxes represent median value while the whiskers minimal and maximal value (*n* ≥ 8). Statistical significance to control is indicated by either *p* ≤ 0.01 (**) *p* ≤ 0.001 (***) or *p* ≤ 0.0001 (****). Statistical significance was determined using One-way ANOVA with Dunnett’s multiple comparison test

In the case of fat body and muscle tissues, significant differences were noted only for the glucose level in muscle tissue (F = 6.861, *p* = 0.0002) (Fig. 2). The level of this sugar increased in muscles during cold stress by 57% (Dunnett’s post-hoc, *p* = 0.0006) and remained elevated by 70% and 61% during 3 and 8 h of recovery, respectively (Dunnett’s post-hoc, *p* = 0.0005 and *p* = 0.0024, respectively) (Fig. 2B). In the fat body, the level of glucose did not differ significantly from the control in all tested conditions (F = 0.5525, *p* = 0.6981) (Fig. 2D). Additionally, the glycogen level did not change significantly in either tissue under all experimental conditions.

**Fig. 3:**
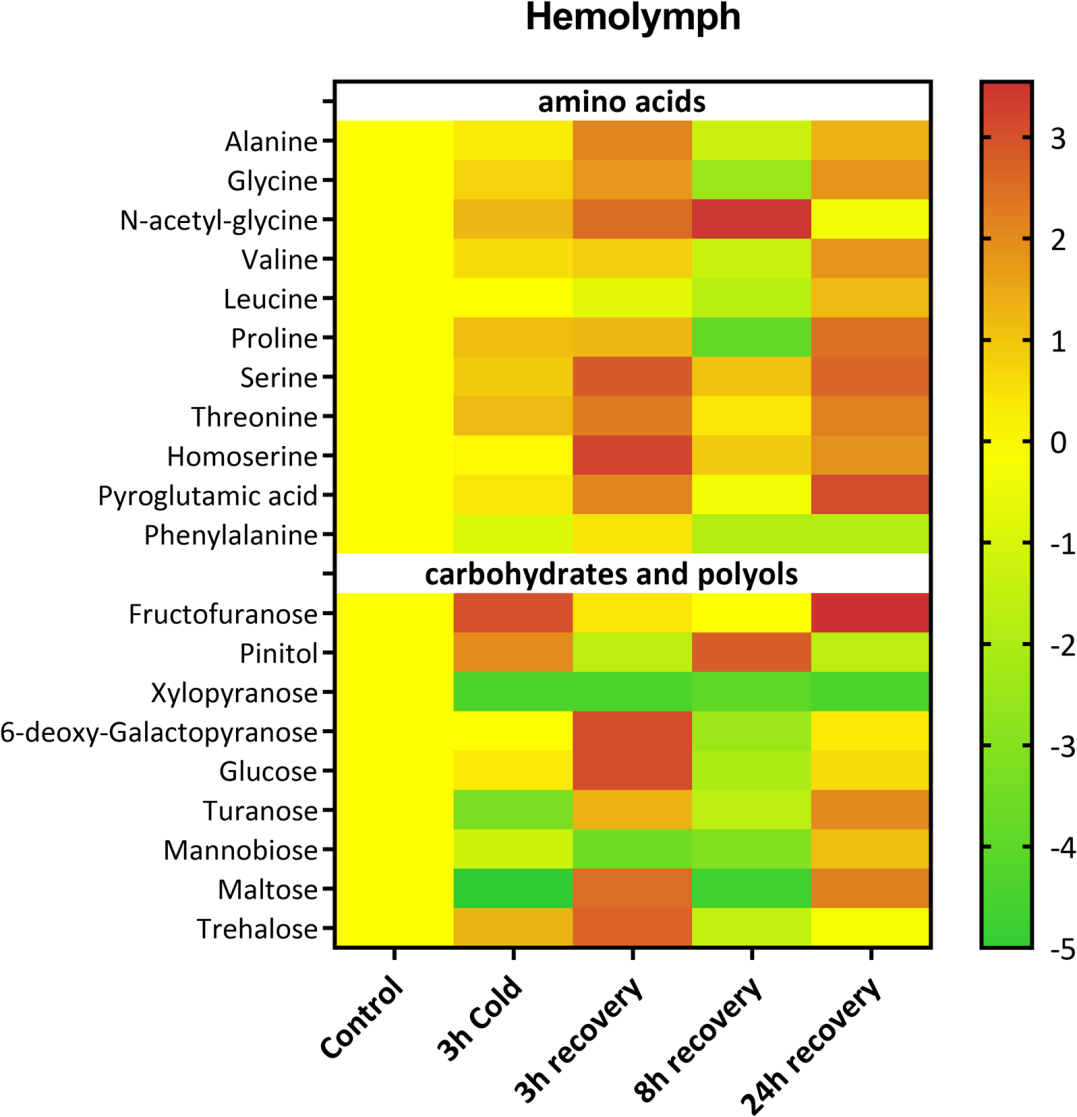
Changes in the level of amino acids, carbohydrates, and polyols in the hemolymph of *G. coquereliana* after cold stress and recovery periods. Data are presented as Log2FC values, with the scale indicating higher (value > 0) or lower (value < 0) metabolite levels compared to the control (value = 0). White boxes indicate that the level of a metabolite was below the limit of detection. The statistics for the figure are presented in Table S1

In the hemolymph, we identified 11 amino acids and 9 carbohydrates. The level of almost all the amino acids increased after cold stress, after 3 h recovery and after 24 h recovery (Fig. 3). The only amino acid which level decreased significantly during cold stress was phenylalanine, which level dropped by 44% (Dunett’s post-hoc, *p* = 0.0005) compared to control. After 3 h recovery the only amino acid with lowered levels was leucin which decreased by 39% (Dunett’s post-hoc, *p* = 0.0079). Interestingly, after 8 h recovery the level of 6 aa (Ala, Gly, Val, Leu, Pro, Phe) decreased substantially compared to control, while the Serine, Homoserine and *N*-acetyl-glycine increased substantially, with the latter increasing during that time over 11-times (Dunett’s post-hoc, *p* < 0.0001) (Fig. 3 and Tab. S1 in which statistics for every compound can be found).

For the carbohydrates, during cold stress the level of fructose, pinitol, glucose and trehalose increased, while turanose, mannobiose, xylopyranose and maltose decreased. During the recovery phase, most profound changes can be observed after 3 h at which the level of five carbohydrates (6-deoxy-galatopyranose, glucose, turanose, maltose and trehalose) significantly increased (for all Dunett’s post-hoc, *p* < 0.0001) while the others decreased substantially and at 8 h recovery when the level of pinitol only increased (Dunett’s post-hoc, *p* < 0.0001), while the levels of other carbohydrates decreased significantly, compared to control (Fig. 3 and Table S1 in which statistics for every compound can be found).

### The effect of cold stress on triglycerides and glycerol level

**Fig. 4:**
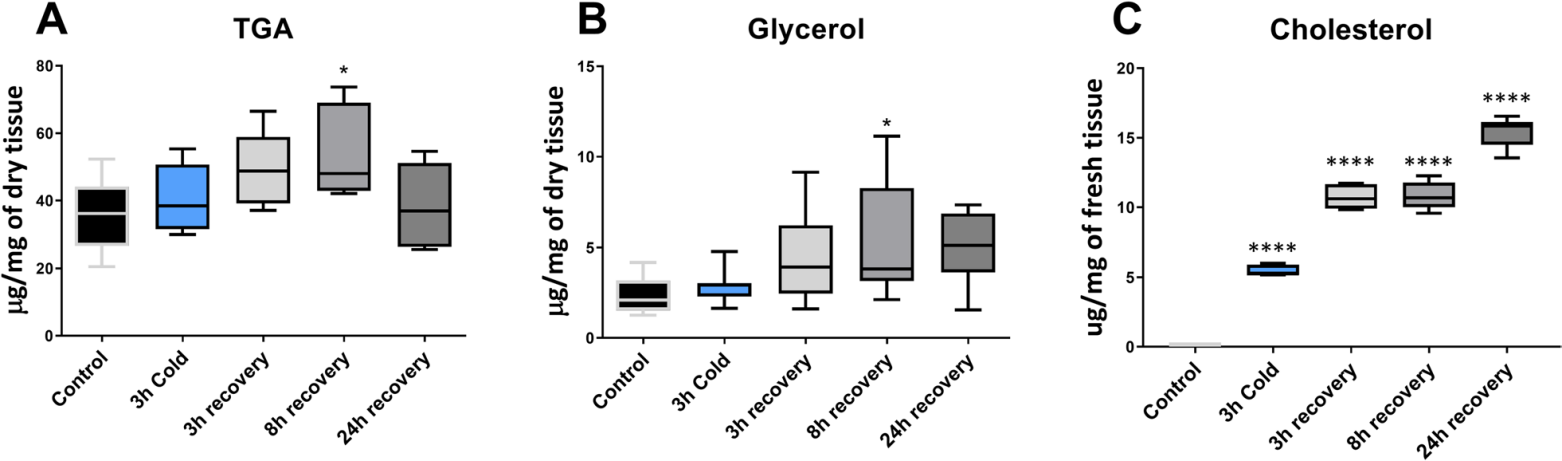
Changes in triglyceride acids (**A**, TGA), glycerol (**B**) and cholesterol (**C**) in the fat body of *G. coquereliana* subjected to cold stress (4 °C) for 3 h and in recovery (3h, 8 h and 24 h) after cold stress. The centre line in boxes represent median value while the whiskers minimal and maximal value (*n* = 8). Statistical significance to control is indicated by *p* ≤ 0.05 (*) and *p* ≤ 0.0001 (****)*.* Statistical significance was determined using One-way ANOVA with Dunnett’s multiple comparisons test

The treatments had significant effect on the level of triglycerides (F = 3.640, *p* = 0.0139), glycerol (F = 3.15, *p* = 0.0259) and cholesterol (F = 279.4, *p* < 0.0001) in the fat body tissue. In case of TAG the level increased after 8 h recovery by 50%, (Dunnett’s post hoc, *p* = 0.0121, Fig. 4A).

In case of glycerol, the statistically significant changes were only observed 8 h after cold stress, with an increase by 131% (Dunnett’s post hoc, *p* = 0.0230) (Fig. 4B).

The level of cholesterol increased significantly in all experimental variants, from 4637% during cold stress to 13,381% during 24 h recovery period (Dunnett’s post hoc, all p-values < 0.0001) (Fig. 4C and Table S1).

### Changes in metabolites caused by cold stress

**Fig. 5:**
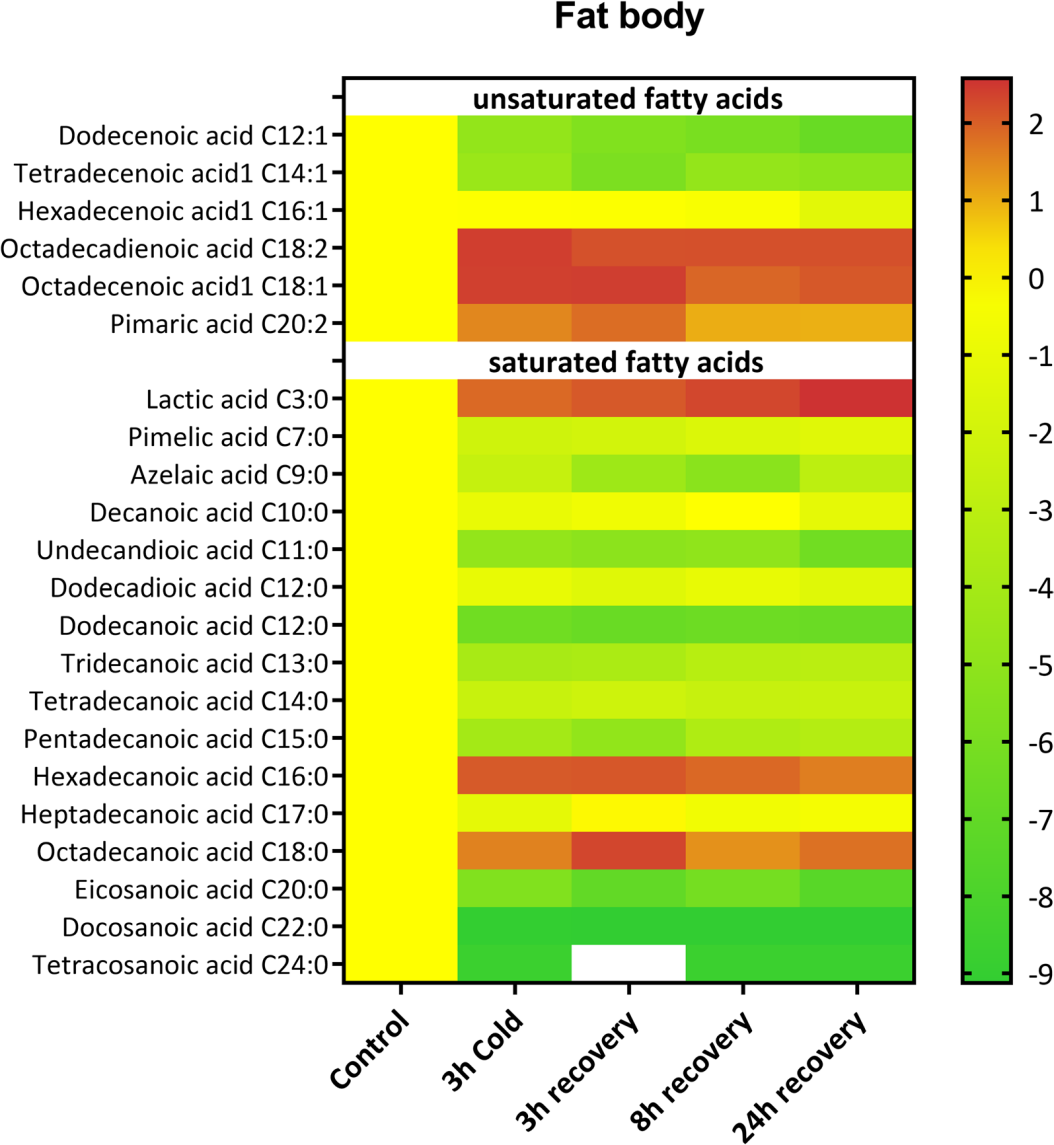
Changes in the levels of unsaturated and saturated fatty acids in the fat body of *G. coquereliana* after cold stress and recovery periods. Data are presented as Log2FC values, with the scale indicating higher (value > 0) or lower (value < 0) metabolite levels compared to the control (value = 0). The white box indicates that the level of a metabolite was below the limit of detection. The statistics for the figure are presented in Table S1

In fat body tissue, we identified 6 unsaturated fatty acids and 16 saturated fatty acids whose levels were altered during the stress and recovery periods (Fig. 5). The data revealed that the level of unsaturated fatty acids (UFAs) changed significantly in all the experimental variants compared with the control group. The levels of four UFAs (C16:1, C18:2, C18:1 and C20:2) increased at all time points (Fig. 5 and Fig. S5). In the case of C12:1 and C14:1, their levels decreased during both cold stress and the whole recovery period.

In the case of saturated fatty acids (SFAs), the three SFAs presented opposite accumulation patterns compared with those of all the other SFAs identified in the FB. Compared with those in the control, lactic acid (C3:0), hexadecanoic acid (C16:0) and octadecenoic acid (C18:0) significantly increased during cold stress as well as during the whole recovery period (Fig. 5, Fig. S6 and Tab. S1 in which statistics for every compound can be found). The rest of the identified SFAs decreased during cold stress, and the recovery with only tetracosanoic acid (C24:0) decreased to undetectable levels after 3 h of recovery.

**Fig. 6:**
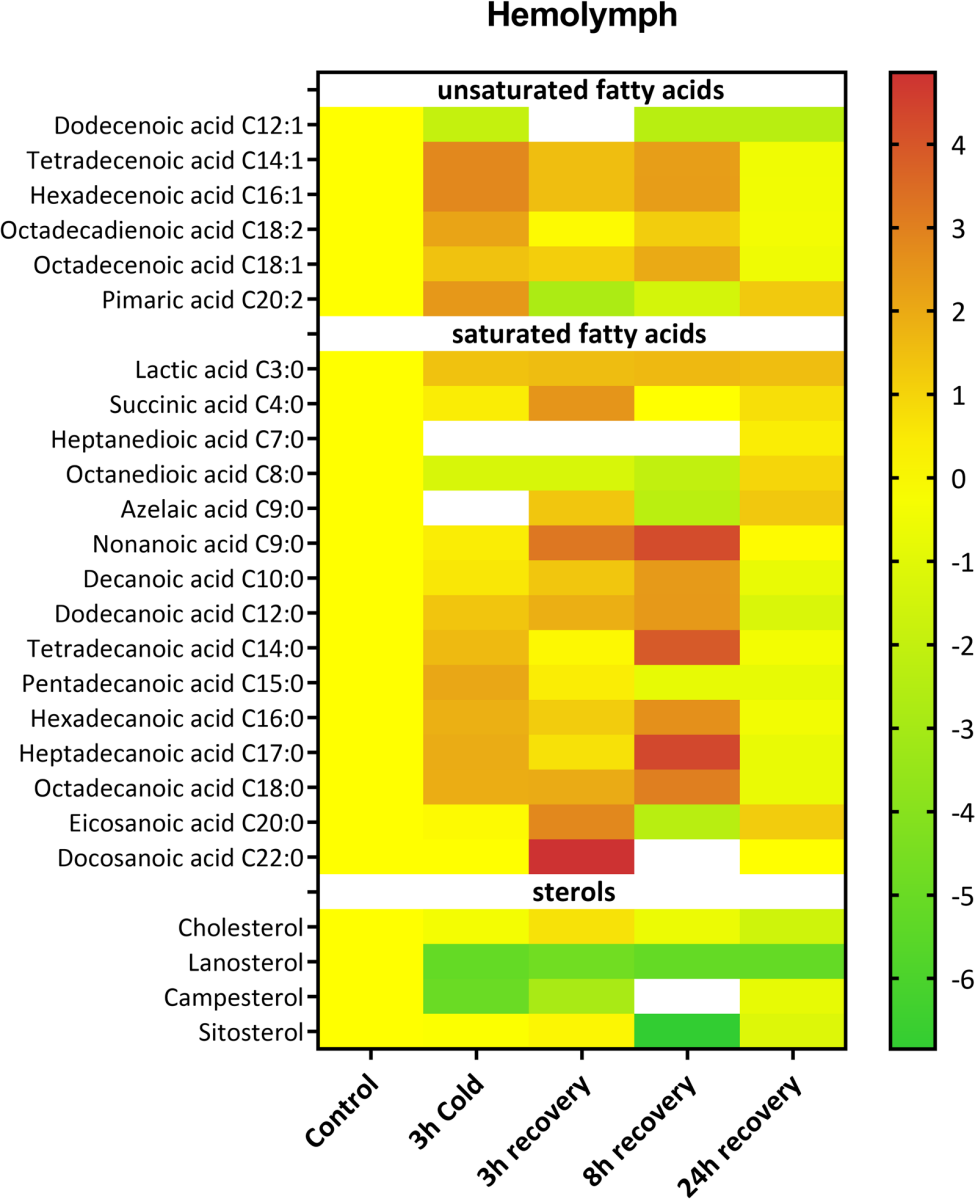
Changes in the levels of sterols and unsaturated and saturated fatty acids in the hemolymph of *G. coquereliana* after cold stress and recovery periods. Data are presented as Log2FC values, with the scale indicating higher (value > 0) or lower (value < 0) metabolite levels compared to the control (value = 0). The white boxes indicate that the level of a metabolite was below the limit of detection. The statistics for the figure are presented in Table S1

In the hemolymph, we identified six UFAs, 15 SFAs and four sterols whose levels changed during the cold stress and recovery periods (Fig. 6, Table S1). The levels of four UFAs, namely, C14:1, C16:1, C18:1 and C18:2, increased during cold stress and, after 3 h and 8 h, decreased to below the control levels after 24 h of recovery (Fig. 6, Fig. S5). Compared with the control, C12:1 was the only UFA whose level decreased in all the treatments. The level of pimaric acid (C20:2) increased significantly after cold stress and after 24 h of recovery.

In the case of SFAs, an overall trend can be observed of an increased level after cold exposure and during the recovery periods after cold exposure (Fig. 6, Fig. S6 and Table S1 in which statistics for every compound can be found). The level of sterols in the hemolymph decreased under all experimental conditions, except for cholesterol, which increased after 3 h of recovery (Fig. S7).

**Fig. 7:**
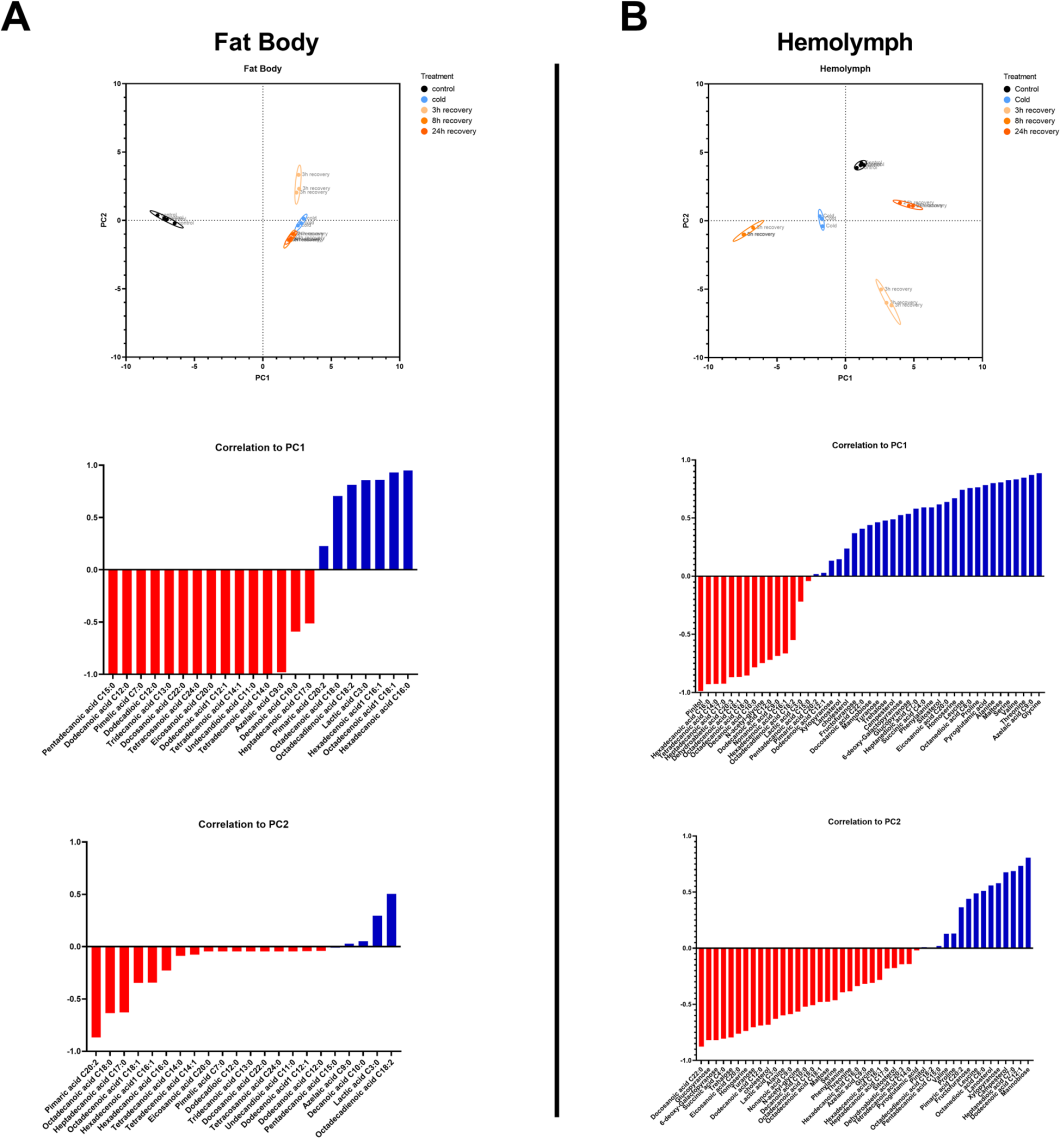
Principal component analyses (PCAs) displaying the first two components (PC1 vs. PC2) based on metabolites identified in fat body (**A**) and hemolymph (**B**) isolated from *G. coquereliana* subjected to cold stress for 3 h and during recovery (3h, 8 h and 24 h recovery) from stress. The first two principal components (PCs) constitute 91.65% of the variability: 81.57% and 10.09% for PCs 1 and 2, respectively, for (**A**), and 73.53% of the variability: 44.69% and 28.84% for PCs 1 and 2, respectively, for (**B**). The metabolite correlations to individual PCs are presented below the respective PCAs, with blue columns denoting positive correlations and red columns indicating negative correlations

In the fat body, the percentage of sterols increased during cold stress and recovery in a time-dependent manner. The level of UFAs in fat body tissue also increased in all the tested groups, whereas the level of SFAs decreased in all the tested groups (Fig. S8A). In the case of the hemolymph, the level of sterols decreased under all conditions but with no visible pattern, with the greatest decrease occurring after 8 h recovery (Fig. S8B). The percentages of both UFAs and SFAs increased after the cold stress and recovery periods (Fig. S8B). The UFA:SFA ratio was greater than that of the control (0.80) in the fat body tissue of all the experimental groups (Fig. 7). The highest ratio occurred after cold stress (1.55, Dunett’s post-hoc, *p* < 0.0001). On the other hand, in the hemolymph, the UFA:SFA ratio was lower than that of the control (1.44) in all the tested groups, with the lowest value occurring after 3 h of recovery (0.92, Dunett’s post-hoc, *p* = 0.0004).

Using the data obtained from the GC‒MS analysis, we performed two PCAs to analyse the tissue-specific metabotypes among all the experimental variants. The first analysis was performed with all the metabolites identified in the fat body tissue, and the second analysis was performed with all the metabolites detected in the hemolymph. In the case of the FB, clear-cut separation along the first principal component (PC1) was observed between control insects and animals subjected to cold and subsequent recovery. The first two PCs explained 91.65% of the variance, with PC1 accounting for 81.57% and PC2 accounting for 10.09% of the cumulative proportion of variance. The most divergent metabotype could be observed in the samples after 3 h of recovery, which was positioned at the lower right part of the graph. To identify the variables (i.e., metabolites) contributing the most to the PCA structure separation, correlations with the PCs were conducted. The metabolites that most positively correlated with PC1 were hexadecenoic acid (C16:1), octadecenoic acid (C18:1) and hexadecanoic acid (C16:0), while pentadecanoic acid (C15:0), dodecanoic acid (C12:0), pimelic acid (C7:0), dodecadioic acid (C12:0), tridecanoic acid (C13:0), docosanoic acid (C22:0) and tetracosanoic acid (C24:0) correlated most negatively (Fig. 7A). Octadecadienoic acid (C18:2) correlated most positively with PC2, while the most negative correlation was observed for pimaric acid (C20:2) (Fig. 7).

In the case of hemolymph, the first two PCs explained 73.53% of the variance, with PC1 accounting for 44.69% and PC2 accounting for 28.84% of the cumulative proportion of variance. Similar to the fat body, the most divergent metabotype for hemolymph was observed at 3 h recovery, and it was positioned in the lower right quadrant of the graph (Fig. 7B). The metabolites that contributed most positively to PC1 were glycine, azelaic acid (C9:0), threonine and valine, whereas pinitol, hexadecanoic acid (C16:0), tetradecanoic acid (C14:0) and heptadecanoic acid (C17:0) correlated most negatively. Similarly, dodecenoic acid (C12:1), heptanedioic acid (C7:0) and campesterol correlated most positively with PC2, while the most negative correlation was observed for docosanoic acid (C22:0), glucose, 6-deoxy-galactopyranose, succinic acid (C4:0) and trehalose (Fig. 7B).

### The effect of cold stress and recovery period on enzyme expression and activity

**Fig. 8:**
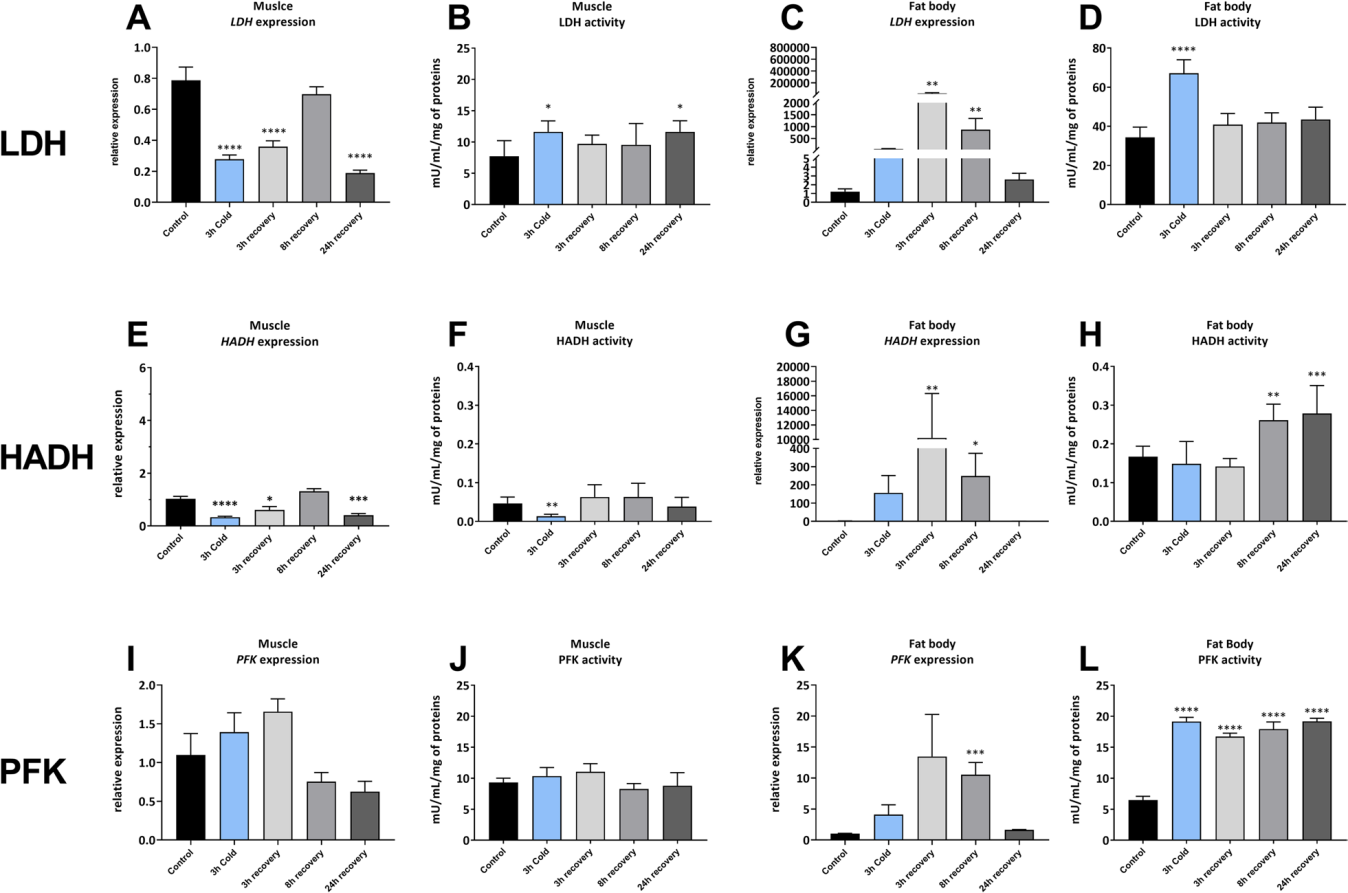
The expression and activity of lactic acid dehydrogenase (LDH), hydroxyacyl-CoA dehydrogenase (HADH) and phosphofructokinase (PFK) in muscle (**A**, **B**, **E**, **F**, **I**, **J**) and fat body (**C**, **D**, **G**, **H**, **K**, **L**) tissues of *G. coquereliana* subjected to cold stress (4 °C) for 3 h and in recovery (3h, 8 h and 24 h) after cold stress. Data represent mean value ± SD (*n* for expression panels = 6; for activity panels = 8). Statistical significance is indicated by *p* ≤ 0.05 (*), *p* ≤ 0.01 (**), *p* ≤ 0.001 (***) or *p* ≤ 0.0001 (****). Statistical significance was determined using One-way ANOVA with Dunnett's (**A**, **B**, **D**, **E**, **H**, **J**, **L**) or Kruskal–Wallis with Dunn’s (**C**, **F**, **G**, **I**, **K**) multiple comparisons test

The treatments affected the *LDH* expression in the muscle tissues (ANOVA, F = 28.74, *p* < 0.0001) (Fig. 8). The expression of *LDH* was significantly changed in almost all timepoints, except for 8 h recovery (Dunnett’s post-hoc, *p* = 0.5247). In all other timepoints, the expression was significantly lowered compared to control: 0.27-fold immediately after cold exposure (Dunnett’s post-hoc, *p* < 0.0001), 0.35-fold after 3 h recovery (Dunnett’s post-hoc, *p* < 0.0001) and 0.18-fold after 24 h recovery (Dunnett’s post-hoc, *p* < 0.0001) (Fig. 8A). The enzymatic activity of the LDH was also affected by treatments (ANOVA, F = 3.627, *p* = 0.0159) and increased after cold treatment by 50% (Dunnett’s post-hoc, *p* = 0.0114). The activity also increased by 50% after 24 h recovery (Dunnett’s post-hoc, *p* = 0.0114) (Fig. 8B).

In the fat body tissue, the expression of *LDH* was also affected by the treatments (Kruskal–Wallis, H = 18.99, *p* = 0.0008), however the significant changes were only observed after 3 and 8 h recovery, at which the expression increased by 19,820-fold (Dunn’s post-hoc, *p* = 0.0026) and 877-fold (Dunn’s post-hoc, *p* = 0.0024), respectively, compared to the control (Fig. 8C). The enzymatic activity of the LDH in the fat body significantly increased only after exposure to cold stress by 95.89% (Dunnett’s post-hoc, *p* < 0.0001) (Fig. 8D).

The *HADH* expression in the muscles was also affected by the treatments (ANOVA, F = 21.55, *p* < 0.0001). It was significantly decreased after cold and 3 and 24 h recovery by 0.34-fold (Dunnett’s post-hoc, *p* < 0.0001), 0.61-fold (Dunnett’s post-hoc, *p* = 0.0110) and 0.41-fold (Dunnett’s post-hoc, *p* = 0.0002), respectively (Fig. 8E). The activity of the enzyme changed only after cold treatment, decreasing by 72% (Dunn’s post-hoc, *p* = 0.0076) (Fig. 8F).

In the fat body, the expression and activity of the *HADH* was affected by the treatments (Kruskal–Wallis, H = 20.02, *p* = 0.0005 and ANOVA, F = 13.14, *p* < 0.0001) (Fig. 8G, H). The gene expression significantly increased after 3 and 8 h recovery by 10 228-fold (Dunn’s post-hoc, *p* = 0.0029) and 249-fold (Dunn’s post-hoc, *p* = 0.0463), respectively. Whereas the activity of the enzyme increased after 8 and 24 h recovery, by 56% (Dunnett’s post-hoc, *p* = 0.0032) and 68% (Dunnett’s post-hoc, *p* = 0.0005), respectively (Fig. 8H).

The expression of the gene encoding PFK was affected by the treatments in the muscle tissue (Kruskal–Wallis, H = 12.34, *p* = 0.0150). However, no significant changes were observed when compared to the control. The activity of the enzyme also did not change significantly in all experimental variants.

In the fat body, the expression was also affected by the treatments (Kruskal–Wallis, H = 15.51, *p* = 0.0038). The expression of *PFK* increased significantly only at one time point, which was after 3 h recovery, 10.5-fold (Dunn’s post-hoc, *p* = 0.0006) (Fig. 8K). However, the enzymatic activity of the PFK increased at all experimental variants (ANOVA, F = 52.86, *p* < 0.0001), compared to control, by 195% after cold stress (Dunnett’s post-hoc, *p* < 0.0001), 158% 3 h after cold (Dunnett’s post-hoc, *p* < 0.0001), 177% 8 h after cold (Dunnett’s post-hoc, *p* < 0.0001), 196% 24 h after cold (Dunnett’s post-hoc, *p* < 0.0001) (Fig. 8L).

## Discussion

Metabolic changes are among the main responses of organisms exposed to environmental stress [104]. In the present study, we explored how *G. coquereliana*, adjusts its metabolism in response to acute cold stress. We focused on crucial metabolic pathways and analysed the levels of metabolites, and the activity and gene expression of key enzymes involved in lipid, carbohydrate, and protein metabolism. As a model, we used the partially freeze-tolerant tropical cockroach *G. coquereliana.* Voituron*, *et al*.* [47] proposed that partial freeze tolerance might be an essential intermediary step in the evolution of freeze tolerance*.* The size of this insect allowed us to conduct tissue-specific analysis, allowing for a more detailed analysis after cold stress and recovery periods.

We observed a significant increase in glucose levels in insect muscles immediately after cold stress. Even more glucose was observed after 3 h of recovery, after which its level slowly decreased at 8 h and stabilized to almost control levels after 24 h of recovery (Fig. 2). Similar trends in *G. coquereliana* cockroach hemolymph were observed for trehalose and fructose. Such increased amounts in response to cold stress have been observed in many insect species [12, 21, 48, 49]. Low-molecular-weight sugars may serve as important energy reserves as well as cryoprotectants by stabilizing proteins and membranes. Moreover, they can reduce the body supercooling point when they accumulate at high concentrations [50]. Trehalose is the principal storage sugar in insects and occurs mainly in hemolymph; trehalose can accumulate in response to environmental stressors, including desiccation, cold or anoxia [51, 52]. It is synthesized mainly from glucose and glycogen via glycolysis and the pentose phosphate pathway, which is an alternative route of glucose mobilisation and a major source of reducing power, NADPH, for biosynthetic processes such as UFA synthesis [50, 53]. In adults of *Drosophila montana*, a freeze avoiding insect, relatively high concentrations of glucose and trehalose are found during the winter. Teets, et al. [29] reported significant glucose mobilization either by glycogenolysis or gluconeogenesis during the freezing and supercooling of *Belgica antartica* [12]. The accumulation of trehalose was observed in winter in the mountain pine beetle *Dendroctonus ponderosae* [24] and *D. immigrans* [22]. It can also be converted back to glycogen and therefore may be related to energy storage functions [54]. However, the amount of glycogen in the fat body and muscle tissues of cockroaches after cold stress and within 3 h of recovery did not change significantly (Fig. 2). Thus, we suppose that the increase in glucose concentration we observed in cockroaches might be a consequence of gluconeogenesis rather than glycogenolysis.

An increase in cryoprotectants such as proline is a part of the adaptive strategy of insects exposed to cold stress. Proline was elevated almost threefold in cockroach hemolymph exposed to cold stress. Other amino acids, such as glycine, threonine, and serine, increased significantly and became even more abundant during the first 3 h of recovery (Fig. 3). The accumulation of amino acids in cold-stressed insects may exert cryoprotective effects through stabilizing cell membranes, protein structures and enzyme activity, even at low concentrations [12, 55–57]. Moreover, proline is an important energy source that maintains ATP homeostasis [58] by scavenging free radicals and buffering the cellular redox potential under stress conditions and is relatively conserved among taxa [59]. Proline accumulation has been reported in the wintering aphid *Cinara tujnafilina* [14], a temperate drosophilid fly, *Chymomyza costata* [60], the fruit fly *D. melanogaster* [61], the beetle *A. diaperinus* [62, 63], the southwestern corn borer *Diatraea grandiosella* and the European corn borer *Ostrinia nubilalis* [64].

The accumulation of amino acids may also arise from the degradation of proteins or the disruption of metabolic pathways [65, 66]. The inability of insects to synthesize most essential amino acids indicates that the relatively high levels of several essential amino acids, e.g., threonine and valine, we observed in the hemolymph of cockroach *G. coquereliana* may be a result of the breakdown of proteins, as suggested in the tropical beetle *A. diaperinus* [63]. This increase was observed in the muscle after recovery and in the fat body tissue immediately after cold stress and during the whole recovery period (Fig. 1). Elevated levels of alanine in cockroach hemolymph may be engaged in stabilizing membrane structures [20, 67] but can also be produced during fermentative glycolysis when oxygen availability is reduced and then serve as a potential source of energy [63, 68]. Together, lactate, glycerol and alanine are the primary precursors for gluconeogenesis, collectively contributing to more than 90% of the total process (Guo et al., 2020). The level of alanine, which also increases during cold stress, can depress the electrical activity of the ventral nerve cord and cause immobility of insects in chill comas [69].

Having shown that the concentrations of glucose and other carbohydrates increased after cold stress, we measured the gene expression and activity of phosphofructokinase. This enzyme plays a crucial role in regulating glycolysis as a rate-limiting enzyme through allosteric inhibition, thereby adjusting the glycolysis rate in response to the energy requirements of the cell [70]. Our results revealed no significant changes in *PFK* gene expression or PFK activity in insect muscle (Fig. 8I, J) but did reveal increased activity of PFK in fat body tissue after cold exposure and during the recovery period (Fig. 8L). The expression of PFK in the fat body exhibited minor variations but consistently showed an increasing trend, with a statistically significant effect observed only after 8 h of recovery. An increase in PFK/glycolytic activity indirectly results in a reduction in glucose concentrations and may lead to the metabolism of other fuel sources, such as lipids and amino acids [71]. The imbalance between glycolysis and the TCA cycle might be related to cold exposure [67]. The increase in intermediates in the TCA cycle, such as succinic acid, observed in cockroaches suggests increased glycolytic processes. The opposite tendency was detected in the aphids *C. tujnafilina* and *B. antartica*, where a shift from glycolysis to the pentose phosphate pathway was observed [14, 72].

Our study revealed that the cockroach *G. coquereliana*, which was exposed to cold stress, entered chill coma; thus, the limited oxygen availability and generation of lactic acid as an end-product of anaerobic conditions to sustain ATP production cannot be excluded [73, 74]. The conversion of pyruvate, the final product of glycolysis, to lactate when the oxygen supply is limited, is catalyzed by lactate dehydrogenase. The large increase in *LDH* expression and activity in the fat body during cold exposure and within the recovery time (Fig. 8C, D) that accompanies an enormous increase in the amount of lactate in the fat body (Fig. 5) confirms these observations. Surprisingly, the expression of *LDH* in insect muscle decreased significantly, which was not reflected in LDH activity (Fig. 8A, B). We suggest that lactate produced in the fat body can be utilized by the better-oxygenated leg muscles through oxidation back to pyruvate, which can then be utilized to fuel the Krebs cycle. It can also be converted to glucose during gluconeogenesis. Reduced enzyme activities, metabolite levels, and metabolism in cockroach muscle under cold stress were previously reported by Lubawy*, *et al*.* [75] and are plausibly the core response to cold stress in this tropical insect. It seems reasonable to suggest that fat body tissue is mainly responsible for the adjustment of *G. coquereliana* to cold stress. However, despite the induction of anaerobic metabolic pathways, insect tolerance to cold stress seems not to be affected by oxygen limitation in cockroach as well as in False codling moth *Thaumatotibia leucotreta* larvae [69].

High LDH activity in insect tissues is predominantly associated with anaerobic metabolism; however, the results concerning LDH activity and lactate levels are sometimes contradictory. The goldenrod gall moth *Epiblema scudderiana* does not increase the level of lactate despite lowered metabolism, in contrast to larvae of the goldenrod gall fly *E. solidaginis,* which accumulate lactate in an anaerobic state during freezing [76]. Low-temperature exposure could also affect LDH activity differently in different tissues, as observed in the silkworm *Philosamia ricini,* where LDH activity decreased significantly in the hemolymph and increased in the fat body [77]. High in vitro LDH activity was recorded in diapausing larvae of the European corn borer *O. nubilalis* at temperatures close to 0 °C [78]. The authors explained that lactate, which can be toxic at high concentrations, might be excreted into the hemolymph and reabsorbed via lactate/pyruvate transporters and reconverted into pyruvate. Another explanation proposed by Uzelac, et al. [78] is that high amounts of lactate in the hemolymph might inhibit the production of lactate in other tissues via a negative feedback loop on LDH [78]. We did not observe a significant increase in lactate in the *G. coquereliana* hemolymph, but its extremely high level in the fat body after cold stress indicates that the cockroach strategy may rely on the production of glucose from lactic acid in the process of gluconeogenesis and using it as cryoprotectant to improve the cold tolerance or/and maintain the energetic status of insects. Although cockroaches went into the chill coma, switched their metabolism in the fat body to anaerobic and suppressed muscle metabolism, no significant decrease in ATP production or the condition of mitochondria was observed [75].

Lipids are the major component of the fat body, representing more than 50% of the dry weight [79]. They are also among the primary targets during the freezing or chilling of insects. Changes in lipid metabolism and lipid modifications linked with cold tolerance include homeoviscous adaptation, which reflects, among others in an increase in the unsaturation of fatty acid carbon chains and/or shortening of fatty acids carbon chain length [80, 81]. The role of unsaturated fatty acids (UFAs), which stabilize cell membranes, in the adaptation of insects to cold has been previously shown in many insects [15, 32, 39]. In the fat body tissue of *G. coquereliana*, we observed high amounts of dodecanoic acid (C12:1) and palmitoleic acid (C16:1), which are typical components of TAG in fat body tissue (Fig. 5). The levels of these fatty acids, together with saturated fatty acid (SFA) 12:0, decreased or did not change after cold stress and recovery. We observed a significant increase in fat body UFAs, mainly linoleic acid (C18:2) and oleic acid (C18:1), as well as two SFAs, palmitic acid (C16:0) and stearic acid (C18:0). Compared with those of the control, the amounts of lauric acid (C12:0), arachidic acid (C20:0) and other saturated fatty acids decreased, which was reflected in a statistically significant increase in the UFA:SFA ratio (Table 1 and Fig. S8). Such increased proportions of unsaturated fatty acids during the winter were observed in several cold-tolerant species, such as *E. solidaginis* and *E. scudderiana*. Moreover, we observed elevated levels of oleic acid (C18:1; Fig. 5), which plays a pivotal role during cold stress, mostly because it is energetically more favourable for manufacturing than are double-bonded fatty acids [57, 82]. Increased levels of oleic and palmitoleic acids were also detected in *O. nubilalis*, whereas linoleic acid was detected in *P. apterus* [83, 84]. Therefore, the observed response here appears to align with the fundamental strategy many species employ to respond to cold conditions.

**Table 1:** The saturated fatty acid (SFA) and unsaturated fatty acid (UFA) ratios in the fat body and hemolymph of *G. coquereliana* after cold stress and recovery period

	Control	3 h Cold	3 h recovery	8 h recovery	24 h recovery
Fat Body	0,80	1,55	1,37	1,33	1,47
		****	****	****	****
Hemolymph	1,44	1,40	0,92	0,73	1,31
		ns	****	****	***

An increase in the UFA:SFA ratio compared with that of the control is marked in red, and a decrease in the UFA:SFA ratio is marked in green. The results were compared to those of the control using One-way ANOVA with Dunnett’s multiple comparison test, **** *p* ≤ 0.0001, *** *p* ≤ 0.001, ** *p* ≤ 0.01, * *p* ≤ 0.05 (*n* = 3–4)

In *G. coquereliana* hemolymph, the composition of free fatty acids (FFAs) is similar to that of other cockroaches, with relatively high concentrations of C16:0, C18:1, C18:2 and C18:0 [85–87]. In our study, almost all the hemolymph fatty acids increased to varying degrees after cold exposure and during recovery. This increase was most visible 3 h after cold stress, except for Heptandioic (C7:0) and Octanedioic (C8:0) acids. Surprisingly, the ratio of UFAs:SFAs in the hemolymph decreased after 3 and 8 h of recovery and did not change immediately after cold stress and 24 h of recovery (Table 1 and Fig. S8). We suppose that the elevated level of SFAs in cockroach hemolymph may be a consequence of remodelling of the FFA pool in the fat body; at the expense of UFAs, more SFAs are released into the hemolymph. It is also possible that some SFAs are released from the cuticle. These lipids seem to play a role in cold tolerance in overwintering insects [82, 88, 89].

In addition to the increasing amounts of fatty acids, we noticed elevated levels of TAG and glycerol in the fat body after 3 h of cold stress and during recovery, but a statistically significant effect was observed only after 8 h of recovery. Insects often accumulate lipid stores within overwintering stages and use them as a source of energy [90]. Our study indicates that *G. coquereliana* does not heavily depend on lipids as an energy source, as their lipid levels increased. This is in contrast to freeze-sensitive *E. scudderiana*, where a decrease in total lipids was observed, suggesting the utilization of reserves to maintain basal metabolism [91]. The significant increase in FFAs immediately after cold stress and during recovery suggests the priming of anabolic processes such as fatty acid synthesis. Most acetyl-CoA, which is converted into fatty acids, is derived from carbohydrates via the glycolytic pathway, which also provides glycerol [92]. In our study, the level of glycerol slowly increased at all experimental time points, with a significant difference after 8 h of recovery (Fig. 4B). An increase in glycerol is one of the mechanisms used by insects to mitigate the effects of cold stress [93, 94]. Glycerol acts as an antifreeze agent, lowers the supercooling point of the organism, and stabilizes membranes and proteins [82, 95, 96]. The increase in glycerol levels may stem from the transformation of TAG, but in our study, this was apparently not the case. Therefore, further research is needed to investigate this phenomenon more thoroughly, for example, with stable isotope tracers.

Surprisingly, high amounts of cholesterol (Fig. 4C), which cannot be synthesized de novo by insects [97], appeared in the fat body tissue of *G. coquereliana* immediately after cold stress and even more during recovery. Cholesterol plays a crucial role in rigidifying cell membranes [81]. At low temperatures, cholesterol interferes with the regular packing of fatty acids, preventing the membranes from solidifying and maintaining the requisite level of fluidity. Considering that, in insects, sterols such as cholesterol originate mainly from the diet and that the feeding behaviour of cockroaches is strongly reduced during cold stress, we suppose that cholesterol can be obtained from the food remaining in the insect gut. The possibility that it can be transferred from the cuticle cannot be excluded also [98]. Immediately after insects recover from chill coma, they start feeding; thus, increased cholesterol levels during the recovery period may arise from food. Surprisingly, we identified transcripts for cholesterol synthesis intermediates in cockroaches, and their levels increased significantly during cold stress compared with those in the control (data not shown), which requires further study.

The insect response to cold stress occurs at different levels, including the activity and expression of enzymes involved in lipid metabolism. In our study, we analysed hydroxyl-CoA dehydrogenase, an enzyme involved in fatty acid β-oxidation to generate acetyl-CoA. Generally, the activity of this enzyme is much greater in the fat body than in the muscle (Fig. 8F, G), which is not surprising because this tissue functions as a metabolic centre and lipid store [99]. In the case of the other enzyme activities described above (PFK, LDH), a decrease in *HADH* expression and HADH activity in muscle was also noted (Fig. 8E, F). A similar response was observed in *E. solidaginis,* which preserves lipid reserves in parallel [18]. Different processes occur in fat body tissue, including significant increases in *HADH* expression and HADH activity during the recovery period. However, it was not correlated with the depletion of lipid reserves in this tissue. Similar effects were observed in *E. scuderriana* [91]. Acetyl-CoA can promote glycolysis through nonenzymatic acetylation, which enhances lactate formation [100]. Moreover, acetyl-CoA is a key intermediate for the interconversion of fats and carbohydrates, which indicates that HADH is engaged in maintaining energetic homeostasis in insects exposed to cold stress [101].

In summary, the response to cold stress involves marked variations in several metabolic pathways, which likely allows the maintenance of cellular homeostasis in the tropical cockroach *G. coquerelina.* This endemic insect of Madagascar has evolved mechanisms that enable it to cope with daily/seasonal exposure to low temperatures. The fat body, hemolymph, and muscle of *G. coquereliana* exhibit distinct strategies in response to cold stress, which represent forms of adaptation. However, under unfavourable temperature conditions, the fat body appears to play a pivotal role in energy management, as it is a centre of hormonal and nutritional regulation [102]. It appears that *G. coquereliana* exhibits characteristics of both freeze-intolerant and freeze-tolerant insects. While we do not have direct evidence for a directional evolutionary adaptation, the species shows signs of moderate freeze tolerance [37], which could be an important step toward full freeze tolerance. This partial freeze tolerance could indeed be an adaptive strategy for surviving in environments with brief cold periods, such as those found in the southern hemisphere or tropical high mountains [103]. To generalize our results, further experiments should also be conducted with wild individuals collected from the field, as our insects, like all species reared in laboratory conditions, have likely become somewhat adapted to our specific rearing environment. Secondly, *G. coquereliana* may employ behavioral adaptations for thermoregulation, such as burrowing or aggregating in groups, to enhance its survival at low temperatures. Investigating the complementary roles of molecular and behavioral mechanisms in thermoregulation could provide deeper insights into the survival strategies of *G. coquereliana* under low temepratures. Overall, despite the challenges posed by cold stress to the tropical cockroach *G. coquereliana* in maintaining metabolic homeostasis, these insects demonstrate remarkable physiological strategies to cope with adverse environmental conditions such as low temperatures.

## Supplementary Information

Supplementary Material 1

Supplementary Material 2

## Data Availability

The data presented in the study are available on reasonable request from the corresponding author.

## Abbreviations

TGA: Triacylglycerides
UFA: Unsaturated fatty acids
SFA: Saturated fatty acids
PFK: Phosphofructokinase
HADH: Hydroxyacyl-CoA dehydrogenase
LDH: Lactic acid dehydrogenase
HSPs: Heat shock proteins
SCP: Supercooling point
PBS-T: PBS with 0.05% Tween buffer
GC–MS: Gas chromatography-mass spectrometry
