# Supplementary material for: Metabolism dynamics in tropical cockroach during a cold-induced recovery period
Source: Biol Res. 2025 Jun 14;58:40. doi: 10.1186/s40659-025-00621-6 (PMC12166600; doi:10.1186/s40659-025-00621-6)
Supplement: Supplementary file 1 — Supplementary Material 1 [file 40659_2025_621_MOESM1_ESM.docx]

Supplementary Information to:

**Metabolism Dynamics in Tropical Cockroach during a Cold-Induced Recovery Period**

Chowański S. ^1^, Lubawy J. ^1*^, Paluch-Lubawa E. ^2^, Gołębiowski M. ^3^, Colinet H. ^4^, Słocińska M.^1^

1. Department of Animal Physiology and Developmental Biology, Adam Mickiewicz University, Poznań, Poland
2. Department of Plant Physiology, Adam Mickiewicz University, Poznań, Poland
3. Laboratory of Analysis of Natural Compounds, Department of Environmental Analysis, Faculty of Chemistry, University of Gdańsk, Gdańsk, Poland
4. ECOBIO – UMR 6553, Université de Rennes 1, CNRS, Rennes, France

*Corresponding author

[j.lubawy@amu.edu.pl](mailto:j.lubawy@amu.edu.pl)


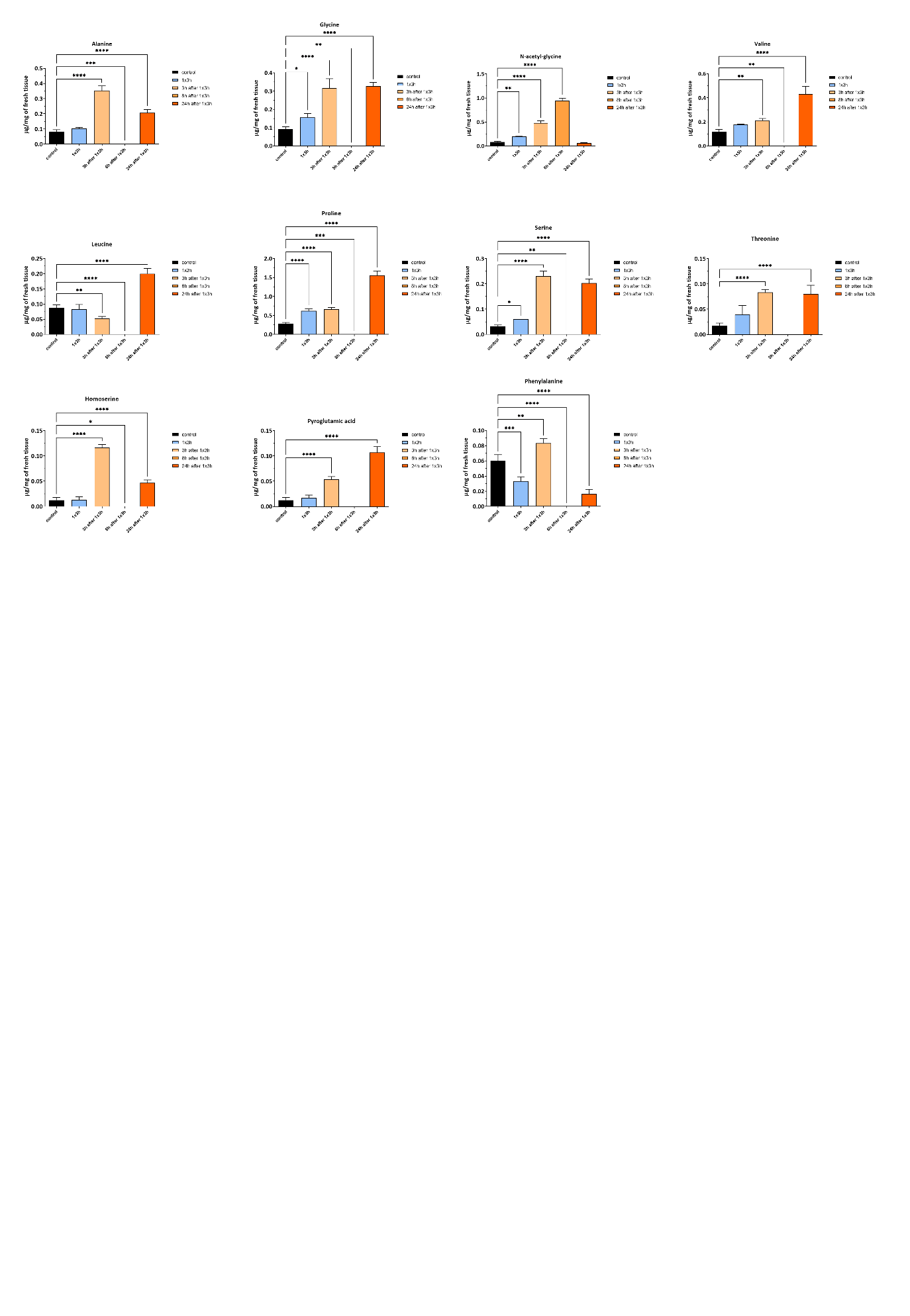


**Figure S1**. Changes in the levels of amino acids in the Hemolymph of *G. coquereliana* subjected to cold stress (4 °C) for 3 h and in recovery (3 h, 8 h and 24 h) after cold stress. The bars represent the mean values ± SDs . Statistical significance to control is indicated by either *p* ≤ 0.01 (**) *p* ≤ 0.001 (***) or *p* ≤ 0.0001 (****). Statistical significance was determined using one-way ANOVA with Dunnett’s multiple comparison test.


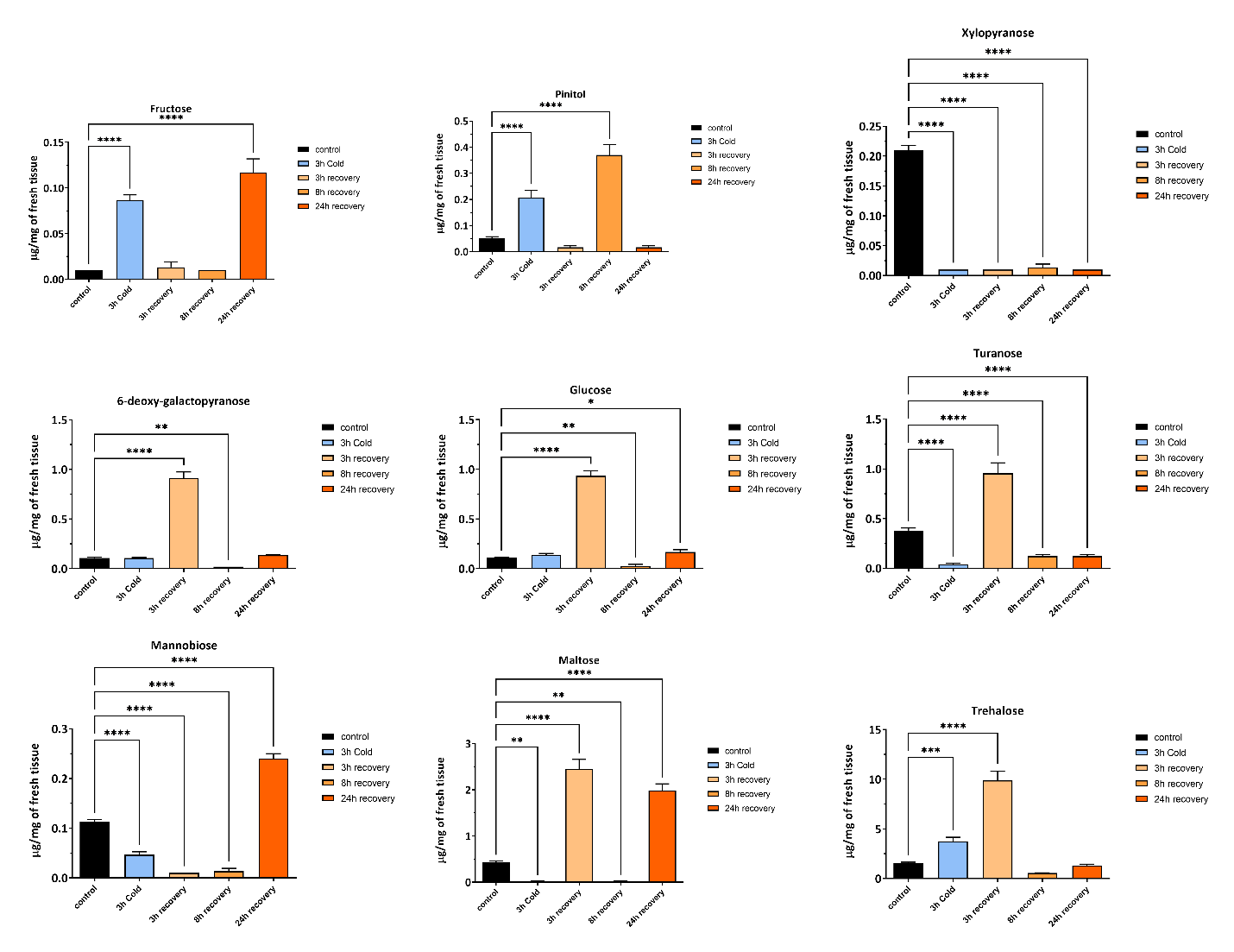


**Figure S2**. Changes in the levels of carbohydrates and polyols in the hemolymph of *G. coquereliana* subjected to cold stress (4 °C) for 3 h and in recovery (3 h, 8 h and 24 h) after cold stress. The bars represent the mean values ± SDs. Statistical significance to control is indicated by either *p* ≤ 0.01 (**) *p* ≤ 0.001 (***) or *p* ≤ 0.0001 (****). Statistical significance was determined using one-way ANOVA with Dunnett’s multiple comparison test.


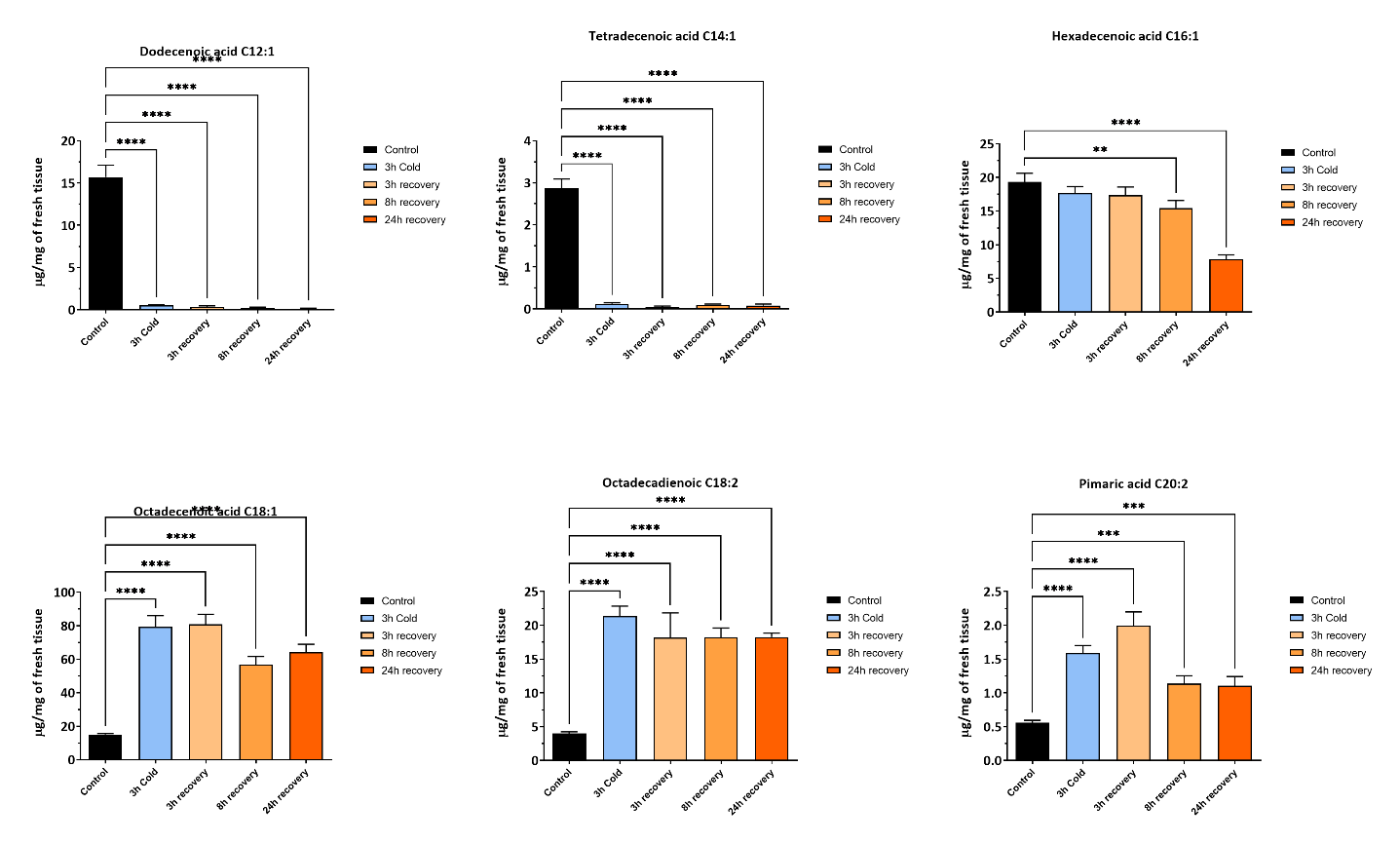


**Figure S3**. Changes in the levels of unsaturated fatty acids in the fat body of *G. coquereliana* subjected to cold stress (4 °C) for 3 h and in recovery (3 h, 8 h and 24 h) after cold stress. The bars represent the mean values ± SDs. Statistical significance to control is indicated by either *p* ≤ 0.01 (**) *p* ≤ 0.001 (***) or *p* ≤ 0.0001 (****). Statistical significance was determined using one-way ANOVA with Dunnett’s multiple comparison test.


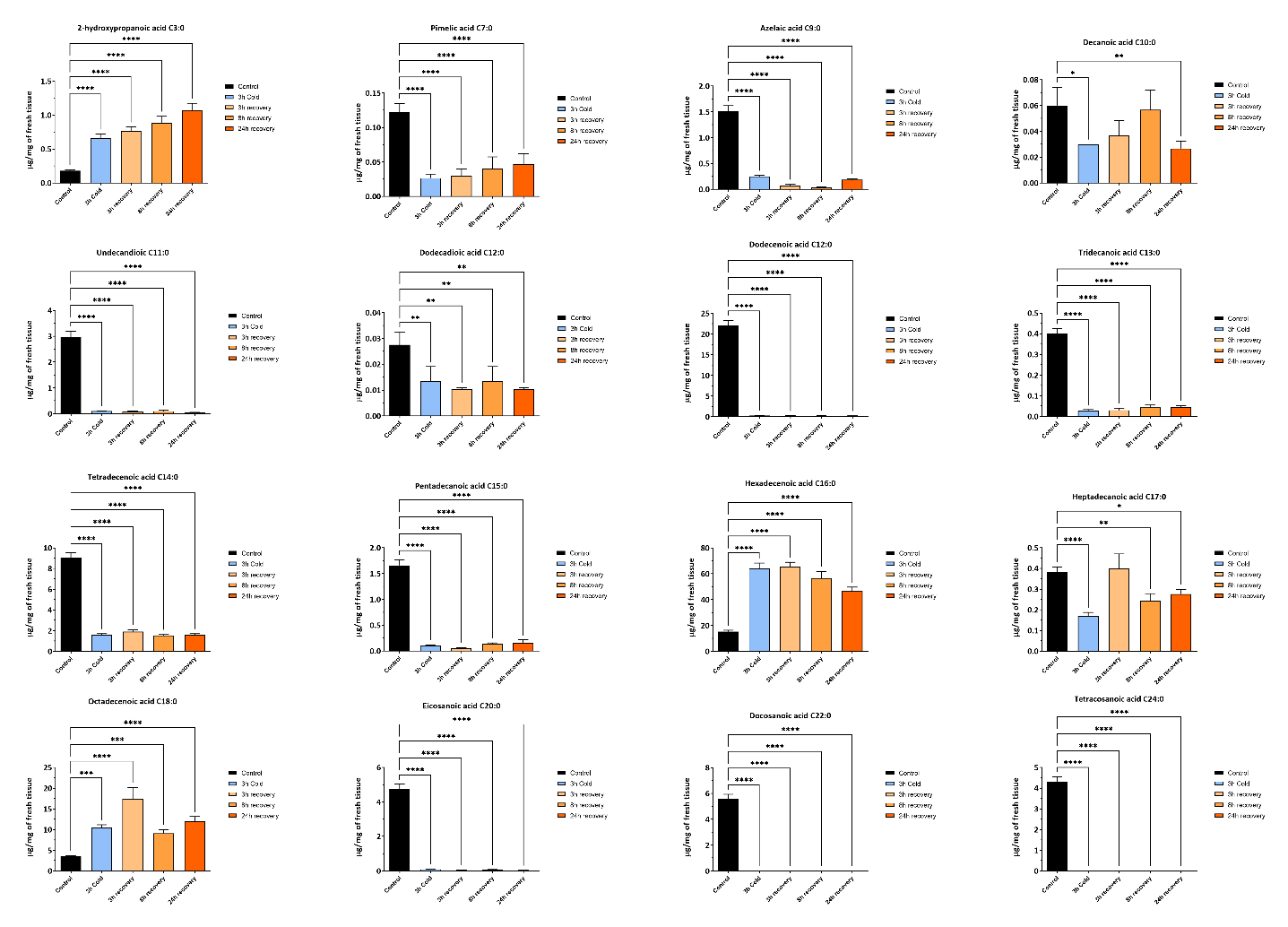


**Figure S4**. Changes in the levels of saturated fatty acids in the fat body of *G. coquereliana* subjected to cold stress (4 °C) for 3 h and in recovery (3 h, 8 h and 24 h) after cold stress. The bars represent the mean values ± SDs. Statistical significance to control is indicated by either *p* ≤ 0.01 (**) *p* ≤ 0.001 (***) or *p* ≤ 0.0001 (****). Statistical significance was determined using one-way ANOVA with Dunnett’s multiple comparison test.


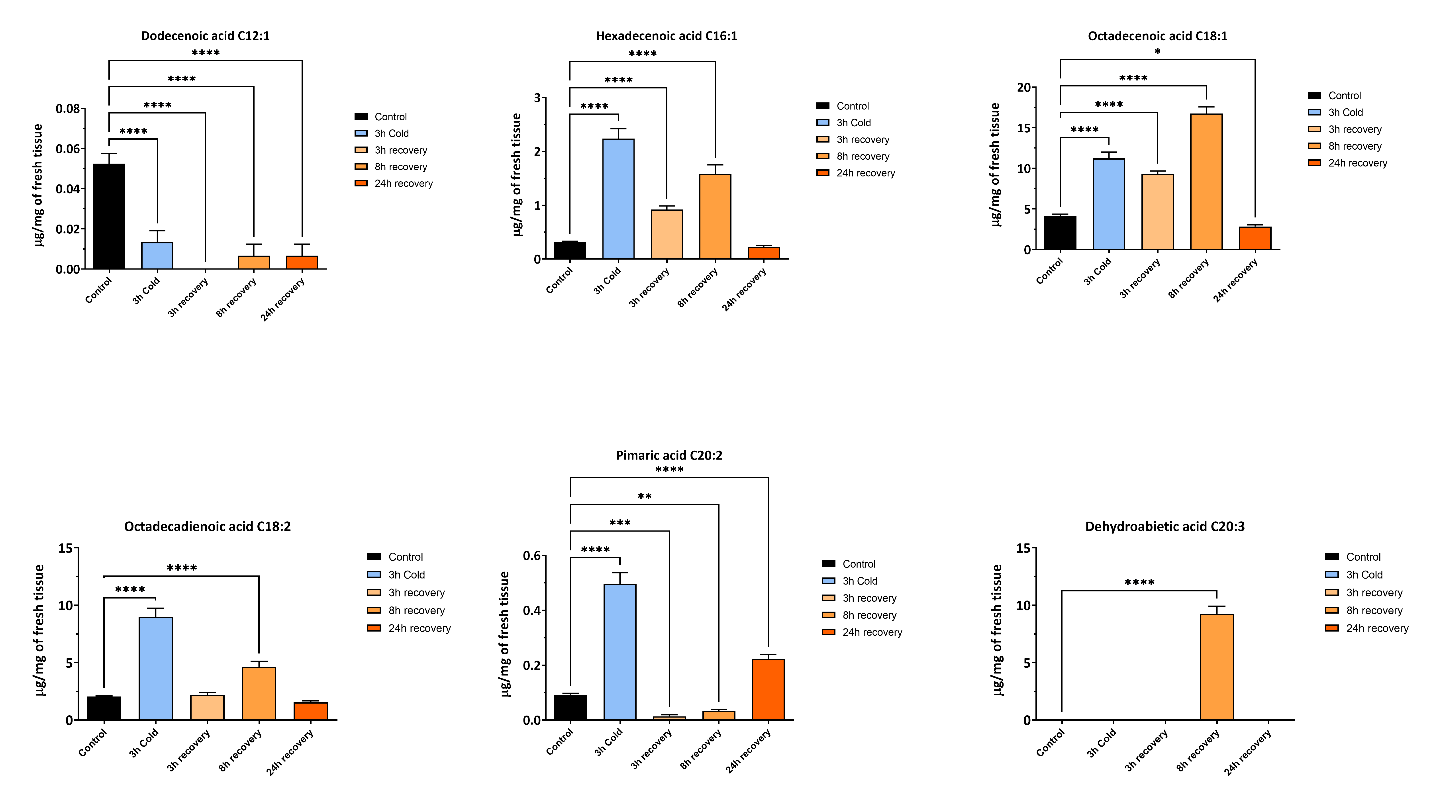


**Figure S5**. Changes in the levels of unsaturated fatty acids in the hemolymph of *G. coquereliana* subjected to cold stress (4 °C) for 3 h and in recovery (3 h, 8 h and 24 h) after cold stress. The bars represent the mean values ± SDs. Statistical significance to control is indicated by either *p* ≤ 0.01 (**) *p* ≤ 0.001 (***) or *p* ≤ 0.0001 (****). Statistical significance was determined using one-way ANOVA with Dunnett’s multiple comparison test.


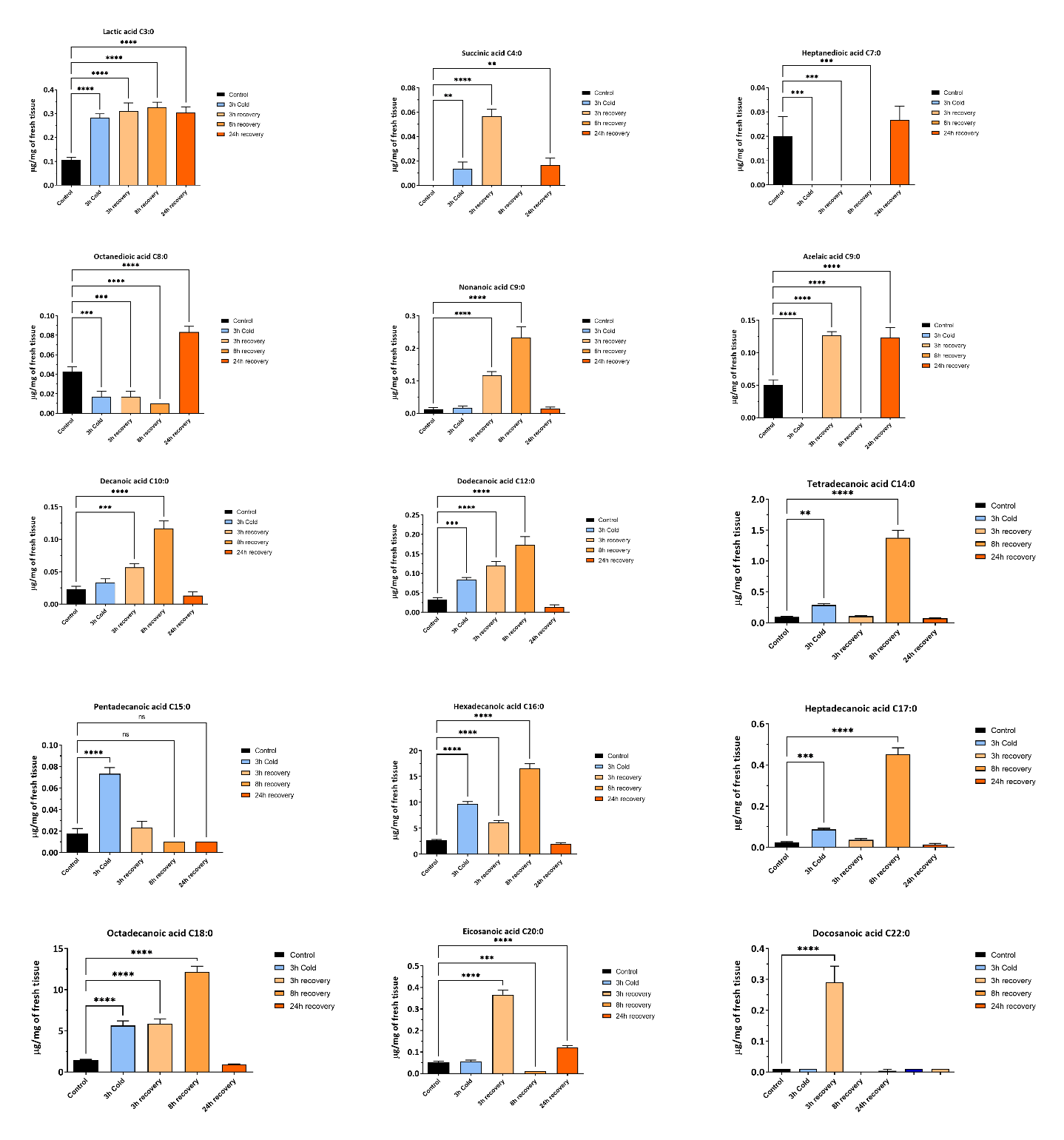


**Figure S6**. Changes in the levels of saturated fatty acids in the fat body of *G. coquereliana* subjected to cold stress (4 °C) for 3 h and in recovery (3 h, 8 h and 24 h) after cold stress. The bars represent the mean values ± SDs. Statistical significance to control is indicated by either *p* ≤ 0.01 (**) *p* ≤ 0.001 (***) or *p* ≤ 0.0001 (****). Statistical significance was determined using one-way ANOVA with Dunnett’s multiple comparison test.


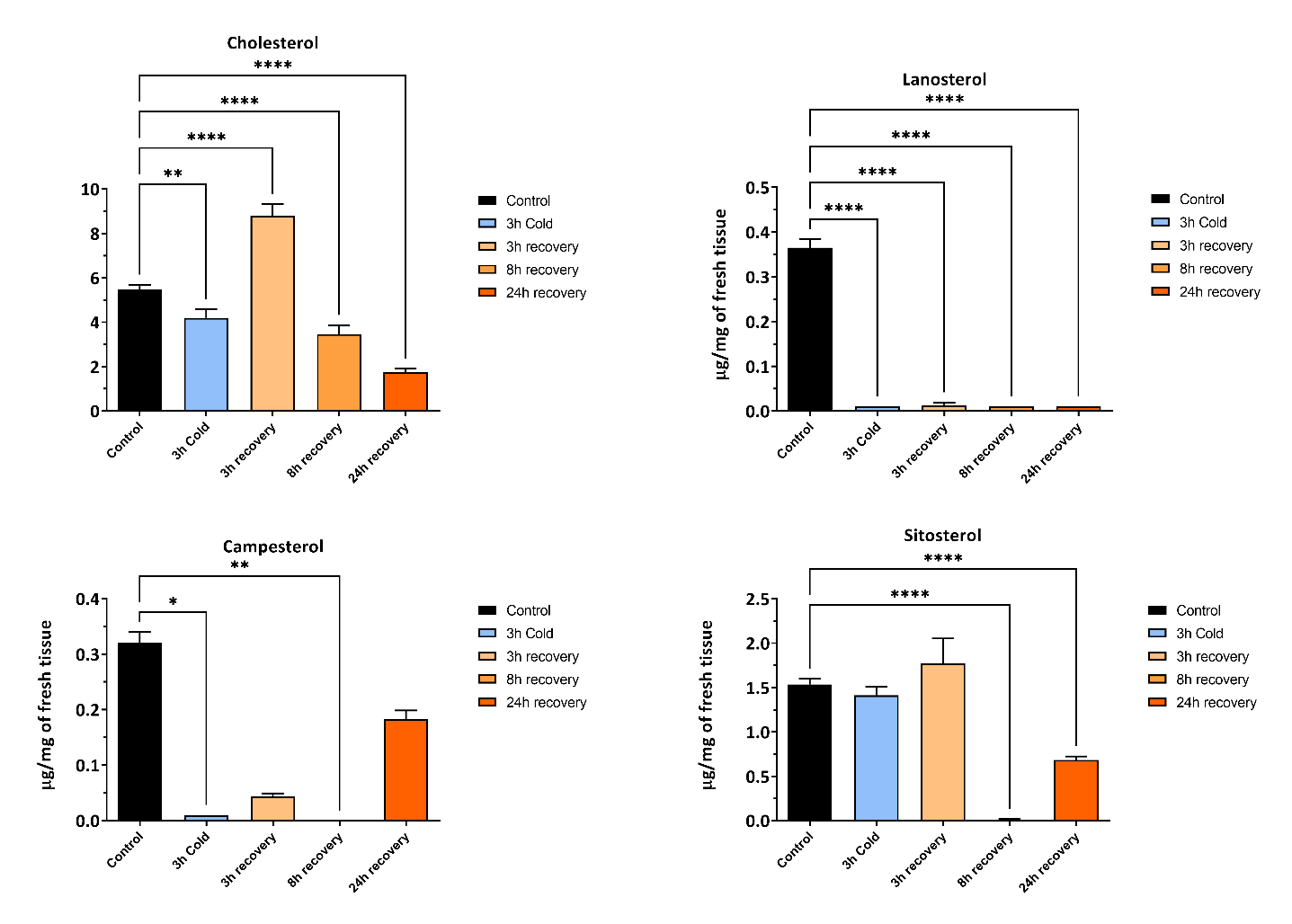


**Figure S7**. Changes in the levels of sterols in the hemolymph of *G. coquereliana* subjected to cold stress (4 °C) for 3 h and in recovery (3 h, 8 h and 24 h) after cold stress. The bars represent the mean values ± SDs. Statistical significance to control is indicated by either *p* ≤ 0.01 (**) *p* ≤ 0.001 (***) or *p* ≤ 0.0001 (****). Statistical significance was determined using one-way ANOVA with Dunnett’s multiple comparison test.


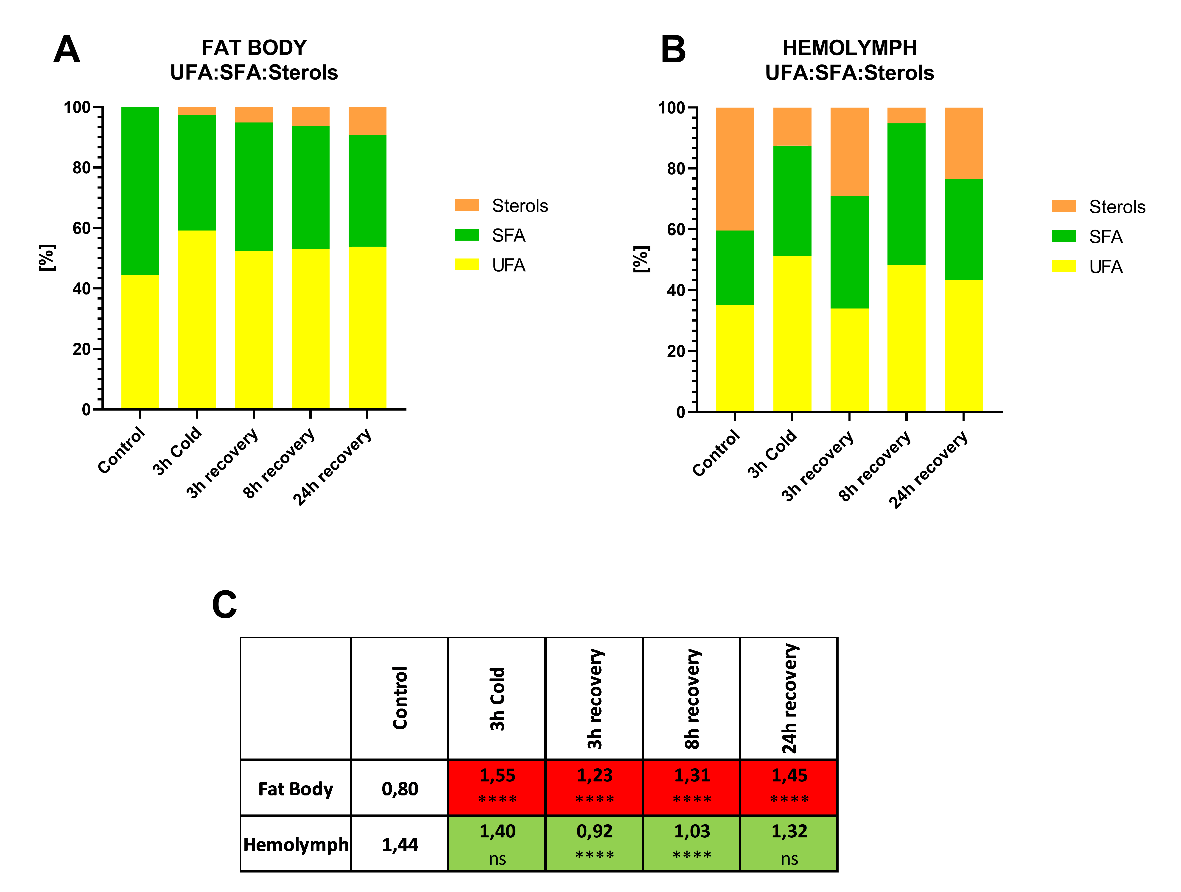


**Figure S8.** The percentage ratio of saturated fatty acids (SFA), unsaturated fatty acids (UFA) and sterols in fat body (A) and hemolymph (B) of *G. coquereliana* after cold stress and recovery periods.


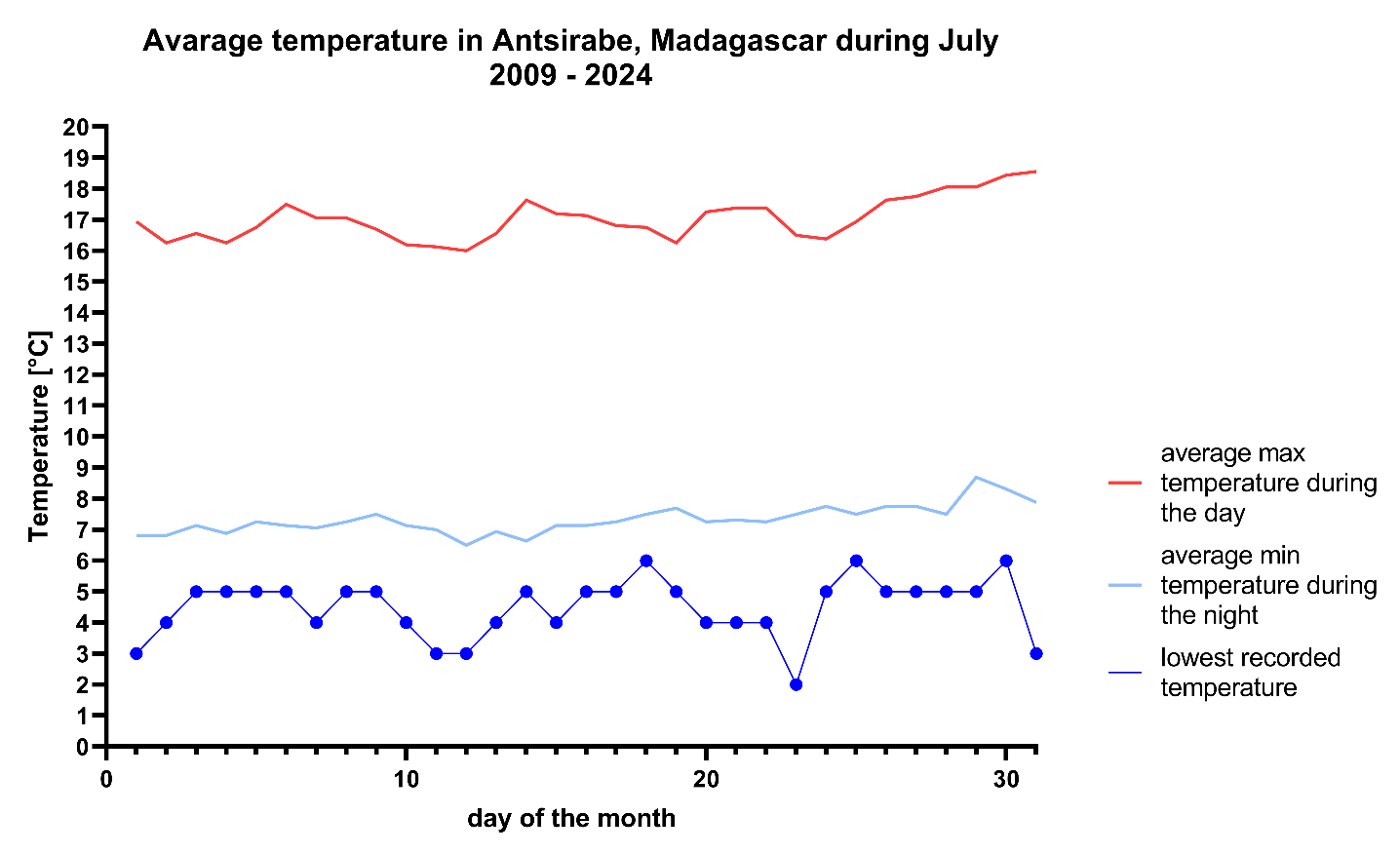


**Figure S9.** Average temperature in Antsirabe (coldest city on Madagascar) during July (coldest month) from year 2009 to 2024. Red line represents average maximal temperature during the day recorded in that period, light blue average minimal temperature recorded during the night and dark blue lowest recorded temperatures in the day over the period mentioned.
